# The TECPR1:ATG5-ATG12 complex conjugates LC3/ATG8 to damaged lysosomes that expose luminal glycans in response to osmotic imbalance

**DOI:** 10.1080/27694127.2025.2476218

**Published:** 2025-05-30

**Authors:** Yingxue Wang, Matthew Jefferson, Maria Ramos, Matthew Whelband, Kristin Kreuzer, Grace Khuu, Michael Lazarou, James Mccoll, James Lazenby, Cynthia B Whitchurch, Paul Verkade, Ulrike Mayer, Thomas Wileman

**Affiliations:** a Norwich Medical School, University of East Anglia, Norwich, UK; b Walter and Eliza Hall Institute of Medical Research, Parkville, Australia; c School of Biochemistry and Molecular Biology, Biomedicine Discovery Institute, Monash University, Melbourne, Australia; d School of Medical Biology, University of Melbourne, Melbourne, Australia; e School of Biological Sciences, University of East Anglia, Norwich, UK; f Quadram Institute Bioscience, Norwich Research Park, Norwich, UK; g School of Biochemistry, Faculty of Life Sciences, University of Bristol, Bristol, UK

**Keywords:** sphingomyelin, Autophagy, galectin 3, LC3/ATG8, lysosome damage, chloroquine, osmotic stress, TECPR1, ATG16L1

## Abstract

Hydrolytic enzymes within lysosomes maintain cell and tissue homoeostasis by degrading macromolecules delivered by endocytosis and autophagy. The release of lysosomal enzymes into the cytosol can induce apoptosis and “lysosome-dependent cell death” making it important for damaged lysosomes to be repaired or removed. Extensive lysosome damage exposes luminal sugars to galectin-dependent autophagy pathways that use ATG16L1:ATG5-ATG12 complex to conjugate LC3/ATG8 to autophagosomes to facilitate removal by lysophagy. Sphingomyelin exposed on stressed lysosomes recruits the lysosome tethering protein TECPR1 (tectonin beta propeller repeat-containing protein) allowing an alternative TECRP1:ATG5-ATG12 complex to conjugate LC3 directly to lysosomes. Here we have used cells lacking ATG16L1 to follow the recruitment of TECPR1, galectin-3 and LC3/ATG8 to lysosomes in response to osmotic imbalance induced by chloroquine. TECPR1 was recruited to swollen lysosomes that exposed sphingomyelin. LC3II was absent from swollen lysosomes but located to small puncta that contained the V-ATPase and LAMP1. The presence of galectin-3 and PI4P in the small LC3 puncta suggested that the TECPR1:ATG5-ATG12 complex conjugates LC3 to lysosome remnants that have ruptured in response to osmotic imbalance.

## Introduction

Lysosomes contain hydrolytic enzymes that degrade macromolecules delivered by endocytosis and autophagy. Lysosomes can protect cells by degrading pathogens and removing of potentially toxic misfolded proteins and can reduce inflammation by degrading cytokine and growth factor receptors, and protein degradation in lysosomes produces amino acids that play an important role in nutrient sensing. Lysosome damage is harmful to cells when hydrolytic enzymes, such as cathepsins released into the cytosol induce apoptosis and “lysosome-dependent cell death” [1]. Lysosomal enzymes can be released into the cytosol through pores formed by an accumulation of poorly digested macromolecules and particulates such as mineral crystals, misfolded proteins and toxic amyloids. Small pores generated during the first few minutes of lysosome permeabilization release Ca^++^ into the cytosol to recruit the endosomal sorting complex (ESCRT). This allows the ESCRT III protein complex to extrude patches of permeabilized membrane into the lumen of the lysosome [2,3]. Ca^++^ release also activates localised generation of phosphatidylinositol-4-phosphate (PI4P) by PI4P kinase which tethers oxysterol-binding protein related proteins (ORPs) that facilitate membrane repair by exchanging PI4P for cholesterol and phosphatidylserine (PS) at endoplasmic reticulum contact sites [4]. Repair is also facilitated by when sites of damage/pore formation are stabilised by assembly of stress granules [5]. If early rounds of repair fail the pores enlarge to expose galactose residues on the N-linked sugars of lysosome membrane proteins present in the lumen of the lysosome to cytosolic galectins. Galectin-8 facilitates autophagy by recruiting autophagy cargo receptor NDP52 [6] and by supressing mTOR activation on the lysosome membrane [7]. Galectins also bind TRIM16 leading to localised protein ubiquitination and assembly of protein complexes to further regulate autophagy and repair [8,9]. Repaired lysosome membranes are rescued through an autophagosome-lysosome regeneration (ALR) pathway that uses kinesin, dynamin and TBC1D15 to extrude lysosome membrane into tubes that form proto-lysosomes [10].

While lysosome repair pathways are being described in detail, much less is known about the fate of unrepaired lysosomes, particularly when extensive damage can lead to rupture and release of lysosomal membranes and enzymes into the cytosol [11]. Galectins and autophagy cargo proteins such as NDP52 and p62 target damaged lysosomes for engulfment by autophagosomes [12]. Once the autophagosomes have sealed, LC3 exposed to the cytosol binds microtubule motor proteins that transport autophagosomes towards lysosomes where LC3 provides a platform for interactions with tethering proteins (HOPS, PLEKHM1) and SNARE proteins (STX17, SNAP29, VAMP8) that promote lysosome fusion and lysophagy [13,14]. Interestingly, several studies document direct conjugation of LC3 to damaged lysosomes [10,15] raising the possibility that the damaged lysosomes may use LC3 to tether to healthy lysosomes. The lysosomal LC3 can also bind the lipid transfer protein ATG2 implicated in lysosome repair [4,16].

Conjugation of LC3 to membranes, also known as ATG8ylation [17] is emerging as a powerful means of targeting membranes to different cellular pathways including membrane degradation and repair. Conjugation involves exposure of a C-terminal glycine residue in LC3 by the ATG4 cysteine protease [18,19–22] followed by conjugation of LC3 to amino groups of phosphatidylethanolamine (PE) or phosphatidyl serine [25] by the E1 and E2-like activities of ATG3 and ATG7, and the E3 ligase-like activity of the ATG16L1:ATG5-ATG12 complex. The ATG16L1:ATG5-ATG12 complex plays a key role during conjugation of LC3 to autophagosomes during canonical autophagy, and during the non-canonical autophagy/CASM pathways, which include the processes of LC3 associated phagocytosis (LAP) and LC3 associated endocytosis (LANDO) that conjugate ATG8/ LC3 to single-membranes in response to raised vacuole pH [23–27]. Proteomic network analysis [28] yeast two hybrid screens and pull downs [29,30] have shown that the ATG5-ATG12 conjugate can also bind the lysosome tethering protein TECPR1 (tectonin beta propeller repeat-containing protein) [30,31] through an ATG5 interaction motif (AFIM) shared with ATG16L1 [32]. The TECPR1:ATG5-ATG12 complex is thought to tether autophagosomes to lysosomes to facilitate autophagosome-lysosome fusion [32], mitophagy and selective autophagy of bacterial pathogens [29]. Recent studies show that TECPR1 also binds sphingomyelin [33–35] exposed early on vacuoles containing bacteria [33] and lysosomes damaged by pore-forming agents [34,35]. Sphingomyelin exposure in the outer leaflet of the vacuole results from changes in lipid packing that occur at early stages in lysosome permeabilization, and these precede the formation of large pores that allow access of galectins to the lysosome lumen [36]. The TECPR1:ATG5-ATG12 complex binds to sphingomyelin where it provides the E3-ligase-like activity able to conjugate LC3 directly to the vacuole to facilitate endo-lysosome repair [34,35]. Here we have used cells lacking ATG16L1 to follow the time course of TECPR1-dependent conjugation of LC3 to lysosomes in response to osmotic imbalance induced by chloroquine. TECPR1 was recruited to swollen lysosomes that exposed sphingomyelin early during osmotic stress, but this was not sufficient to direct LC3 conjugation to the lysosome membrane. LC3 conjugation by the TECPR1:ATG5-ATG12 complex occurred after membrane rupture and exposure of luminal glycans to galectin-3. The results suggest that the TECPR1:ATG5-ATG12 complex conjugates LC3 to lysosome remnants formed after rupture of lysosomes and collapse following loss of osmotic gradients.

## Results

### Chloroquine induces formation of LC3 puncta and LC3II independently of ATG16L1

**Figure 1.**
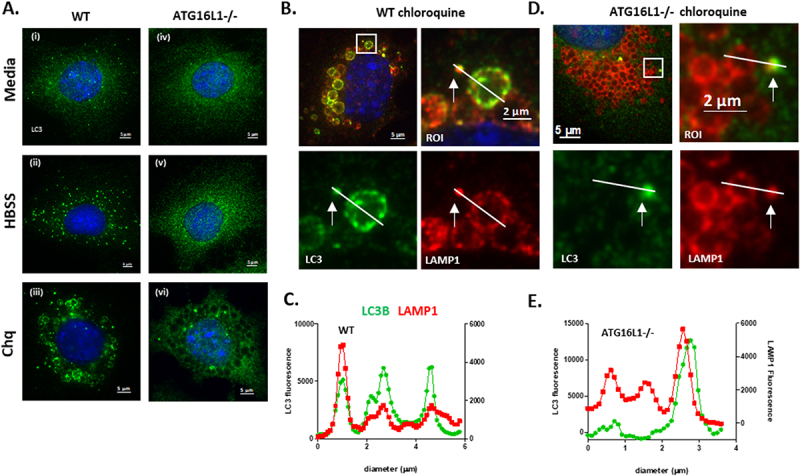
Chloroquine induces ATG16L1 independent recruitment of LC3 to small puncta located close to 505 swollen lysosomes. Panel A. Control (WT) and ATG16L1-/- MEFs were incubated for 2 hours in nutrient media (i and iv), HBSS (ii & v) or nutrient media containing chloroquine (100μM iii and vi) as indicated. Cells were fixed andimmunostained for LC3. Scale bar 5μm. WT MEFs (panel B) or ATG16L1-/- MEFs (panel D) were incubated for 2 hours in media containing chloroquine (100μM). Cells were fixed and immunostained for LC3 and LAMP1. Regions of interest indicated in panels B and D were used to generate line profiles to follow distribution of LC3 and LAMP in small puncta and swollen lysosomes (panel C and E).

**Figure 2.**
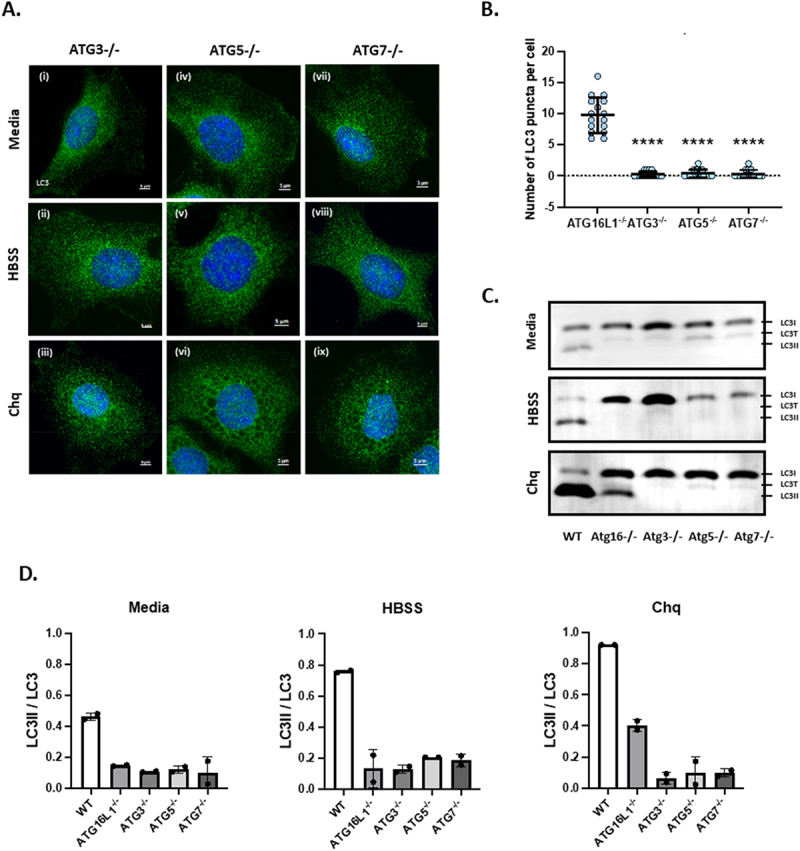
Formation of LC3 puncta and LC3II independently of ATG16L1 requires ATG3, ATG5 and ATG7. Panel A. ATG3-/-, ATG5-/- and ATG7 -/- MEFs were incubated for 2 hours in chloroquine (100 µM) fixed cells were immunostained for LC3. Panel B. LC3 puncta in the indicated cells were counted using Imaris software. Panel C. Cells were incubated as described above and generation of LC3II was assessed by western blot for LC3. Panel D. Densitometric calculation of LC3II/LC3 ratios from duplicate blots.

Figure 1 shows translocation of LC3 to membranes following induction of osmotic imbalance by chloroquine. Panel A compares WT and ATG16L1-/- MEFs and shows that both cell types lack LC3 puncta when cultured in nutrient media (Figure 1A i and iv). The control (WT) MEFS generated LC3 puncta indicative of autophagosomes when starved of amino acids in HBSS (Figure 1A ii) and large LC3 positive vacuoles following induction of osmotic stress by chloroquine (1A iii). Line profiles of LC3 and LAMP1 fluorescence intensity showed that in WT MEFs (Figure 1B,C) LC3 co-localised with LAMP1 in the limiting membrane of swollen lysosomes of approximately 4 μm in diameter and was recruited to small bright LAMP positive puncta of approximately 1.0 μm diameter close to swollen vacuoles. As expected, the ATG16L1-/- MEFs were defective in canonical autophagy indicated by the lack of LC3 puncta after starvation (Figure 1A v) but they were able to generate small bright LC3 puncta of 1 μm diameter in response to osmotic imbalance and raised luminal pH caused by chloroquine (Figure 1A vi). Line profiles of the fluorescence signals (Figure 1D,E, Suppl. Figure 1) showed that the LC3 signal in ATG16L1-/- cells was absent from the limiting membrane of the LAMP1 positive swollen lysosomes but was restricted to the small bright puncta that counter-stained for LAMP1. LC3 puncta induced by chloroquine were not observed in MEFS cells lacking ATG3 (Figure 2A i-iii, Figure 2B), ATG4 (4X KO in HeLa cells, suppl. Figure 2), ATG5 (Figure 2A iv-vi, Figure 2B) or ATG7 (Figure 2A vii-ix, Figure 2B). Western blots (Figure 2C,D) also showed that LC3II was produced in ATG16L1-/- cells incubated with chloroquine. Close inspection of western blots showed two forms of LC3 migrating faster than LC3I. The slower of these two forms was likely to be LC3-T where LC3 is partially degraded by the 20S proteasome and cannot be conjugated to PE [37]. All experiments took care to ensure estimates of LC3II focused on the faster migrating LC3II and not LC3-T. The levels of LC3II induced by chloroquine were lower in ATG16L1-/- cells than seen in WT cells and this reflected the recruitment of LC3II to small puncta rather than the limiting membrane of large swollen lysosomes. As seen for LC3 puncta, chloroquine was unable to induce LC3II in cells lacking ATG3, ATG5 or ATG7. This indicates that the translocation of LC3 to membranes in ATG16L1-/- MEFs in response to chloroquine requires proteolytic processing of LC3 by ATG4 and the E1-like activity of ATG7, the E2-like activity of ATG3 and the E3-like activity of the ATG5-ATG12 conjugate upstream of ATG16L1.

### ATG16L1 independent conjugation of LC3 induced by chloroquine occurs in response to lysosome permeabilization

**Figure 3.**
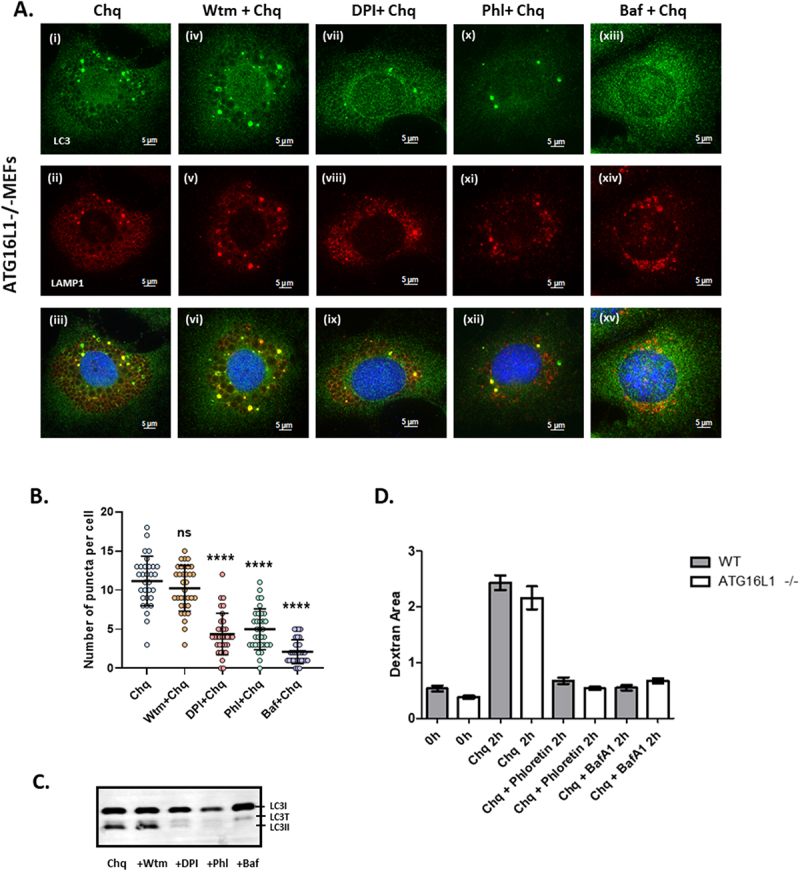
Vacuole swelling in response to osmotic stress induces formation of LC3 puncta and LC3II in ATG16L1-/- cells. Panel A. Atg16L1-/- MEFs were incubated with chloroquine (100 µM) for 2 hours in the presence of wortmannin (100 nM iv-vi) DPI (20 µM vii-ix), phloretin (180 µM x-xii) or bafilomycin (100 nM xiii-xv) as indicated. Cells were fixed and immunostained for LC3 (green) and LAMP1 (red). Panel B. Numbers of LC3 puncta from more than 30 cells incubated as described in panel A were quantified by Imaris^TM^, P-values were calculated using multiple t-test (****P < 0.0001). Panel C. Cells were incubated as described in panel A and generation of LC3II was assessed by western blot for LC3. Panel D. Cells were incubated as described and cross-sectional area (um^2^) of lysosomes containing fluorescent dextran were determined by Fiji. Scale bar 5 μm.

Chloroquine induces an osmotic imbalance in lysosomes that leads to water influx and swelling [38]. A series of inhibitor experiments were used to determine if osmotic imbalance and lysosome swelling, rather than autophagy or raised pH, provided the signal for ATG16L1-independent translocation of LC3 to endo-lysosome compartments. Lysosome swelling in response to chloroquine was followed using cells incubated with fluorescent dextran as a marker for lysosome content. (Suppl. Figure 3A & B). Chloroquine increased the area of lysosomes 4-fold over 2 hours in both WT and ATG16L1-/- MEFs. In parallel experiments LC3 conjugation to small bright puncta was assessed by microscopy (Figure 3A) and quantified by Imaris (Figure 3B) and western blot of cell lysates (Figure 3C). The formation of small LC3 puncta and generation of LC3II in response to chloroquine were unaffected by wortmannin (Figure 3A iv-vi, Figure 3C) and therefore independent of the PI3 kinase activity of vps34 that is upstream of ATG16L1 in canonical autophagy. A possible link with ROS production was indicated by a 60% drop in puncta and LC3II following inhibition of the NADPH oxidase by DPI (Figure 3A vii-ix, Figure 3B,C). A role for osmotic imbalance was tested using phloretin to inhibit water channels [38]. Measurements of lysosome area showed that phloretin reduced lysosome swelling (Figure 3D) and phloretin also reduced numbers of bright LC3 puncta (Figure 3A x-xii, Figure 3B) and formation of LC3II (Figure 3C). Similar results were obtained when lysosome swelling and LC3 conjugation in response to chloroquine were inhibited by bafilomycin (Figure 3A xiii-xv, Figure 3B-D) which raises vacuole pH by inhibiting the v-ATPase but reduces osmotic swelling by slowing the import of protons into vacuoles. The results show that conjugation of LC3 to small puncta required lysosome swelling rather than raised pH, but the lack of direct conjugation of LC3 to the limiting membrane of swollen lysosomes (Figure 1D,E, Suppl. Figure 2) suggests that swelling alone is not sufficient for LC3 conjugation.

**Figure 4.**
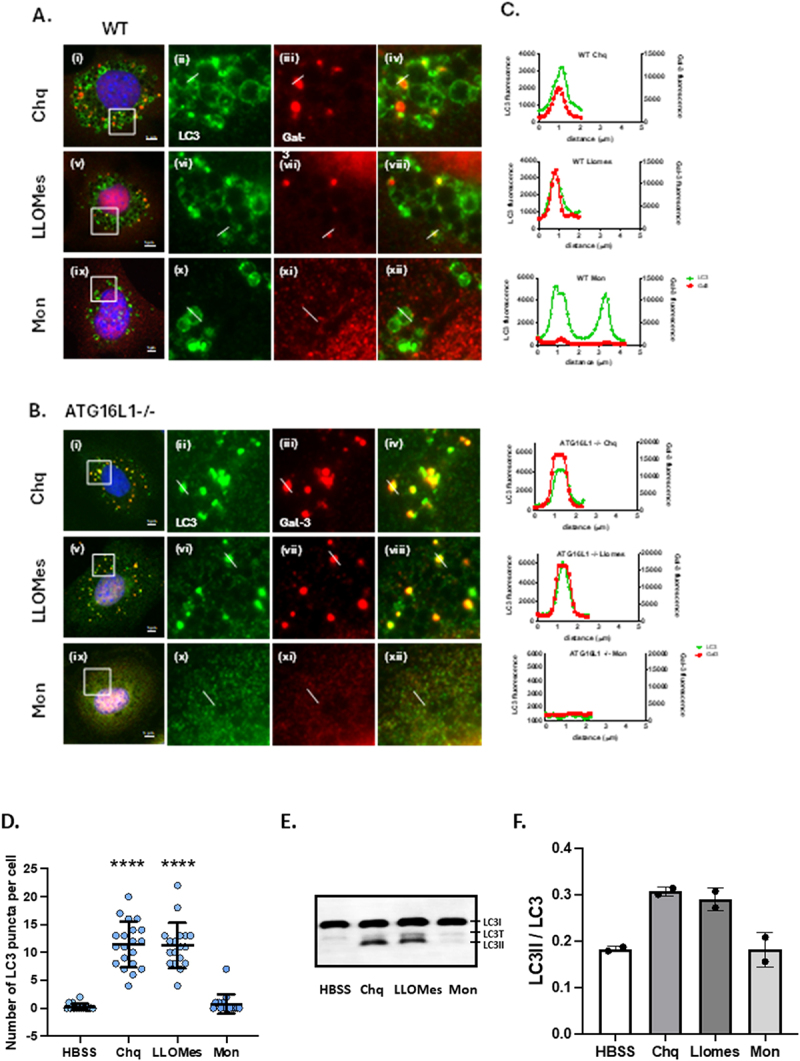
Osmotic stress induces recruitment of galectin-3 to LC3 puncta generated in the absence of ATG16L1. Control (Panel A) and Atg16L1-/- MEFs (Panel B) were incubated for 2 hours in media containing chloroquine (100 µM i-iv), LLOMes (1 mM v-viii) or monensin (100 µM ix-xii). Fixed cells were immunostained for LC3 (green) and galectin 3 (red). Scale bar 5 mm. Panel C. distribution of LC3 (green) and galectin 3 (red) assessed from line profiles. Panel D. Numbers of small (1.0 um dia.) LC3 puncta counted from 20 cells by Imaris. Panel E. LC3II assessed by western blot of cell lysates. Panel F. LC3II/LC3 ratio calculated by densitometry of duplicate blots.

Recent studies show that lysosome permeabilization induced by hydrophobic Leu-Leu-oMe (LLOMe) peptides, or disruption of vacuoles during Salmonella infection, induces ATG16L1-independent translocation of LC3 to vacuole membranes [33–35]. The possibility that osmotic imbalance and swelling induced by chloroquine resulted in lysosome rupture was tested by immunostaining for galectin-3 to identify ruptured membranes that have exposed glycans to the cytosol (Figure 4). In WT MEFS chloroquine generated bright puncta containing galectin 3 (red) which colocalised with LC3 (green) (Figure 4A i-iv). In WT cells the LC3 puncta were close to large swollen vacuoles also staining for LC3 which was likely to have been conjugated to swollen lysosomes by V-ATPase-activated CASM pathways in the WT cells. Corresponding line profiles (Figure 4C) showed that the small puncta contained LC3 and galectin-3. Galectin-3 was absent from the limiting membranes of the swollen vacuoles in WT cells suggesting that they are not damaged/ruptured. In ATG16L1-/- cells (Figure 4B i-iv, Figure 4C) the galectin-3 was again restricted to small bright LC3 puncta, but this time the images showed that both galectin-3 and LC3 were absent from the limiting membrane of swollen lysosomes. It is likely that LC3 is absent from swollen vacuoles in these ATG16L1-/- cells because the CASM pathways that respond to increased vacuole pH are inactive because the cells lack ATG16L1. Osmotic swelling requires membranes to be intact and this is consistent with lack of galectin-3 staining of swollen lysosomes. Once membranes rupture, indicated by recruitment of galectin-3 to exposed glycans, osmotic pressure would be lost allowing the vacuoles to collapse. This would explain why galectin-3 is restricted to smaller puncta rather than larger swollen vacuoles.

The small puncta induced by chloroquine were compared with LC3 puncta generated by LLOMe, where lysosomal dipeptidyl peptidase 1 converts LLOMe into a hydrophobic polymer that generates pores in the lysosome membrane [39]. In WT cells incubated with LLOMe (Figure 4A v-viii) LC3 was recruited to vacuoles and smaller dense puncta, and several of the small puncta were positive for galectin 3 (Figure 3A vii). In ATG16L1-/- cells incubated with LLOMe (Figure 3B v-viii) LC3 was restricted to small galectin–3 positive puncta (Figure 3B vii-viii, line profiles in Figure 4C), again suggesting a link with generation of pores in the lysosome membrane by LLOMe. The requirement for pore formation was also tested using the ionophore monensin which exchanges protons for Na^+^ to raise vacuole pH [40] and induce vacuole swelling but causes less lysosome permeabilization compared to LLOMe [34,35]. It is possible that monensin is less able to permeabilise vacuoles because the concentrations of Na^+^ ions required for exchange with H+ are low in the cytosol [40]. Microscopy (Figure 4A ix-xii) showed that in WT cells monensin generated swollen vacuoles that recruited LC3, presumably through V-ATPase-activated CASM. Line profiles (Figure 4C) showed that the swollen vacuoles lacked galectin-3 and as described previously [35]. ATG16L1-/- cells were unable to recruit LC3 to lysosomes in response to monensin (Figure 4B ix-xii) and monensin failed to generate small galectin-3 or LC3 puncta in either cell type (Figure 3A,B). The absence of galectin puncta following incubation with monensin again suggested, as described previously [34,35] that raised pH was not the primary driver for ATG16L1-independent conjugation of LC3 to membranes. The results from microscopy were supported by counts of small LC3 positive puncta (1.0 μm diameter) in ATG16L1-/- MEFS by Imaris (Figure 4D) which showed increases in puncta in response to chloroquine and LLOMe, but not starvation in HBSS or incubation with monensin. These results correlated with western blots (Figure 4E,F) again showing that LC3II was induced in ATG16L1-/- MEFs by chloroquine and LLOMe, but not monensin.

**Figure 5.**
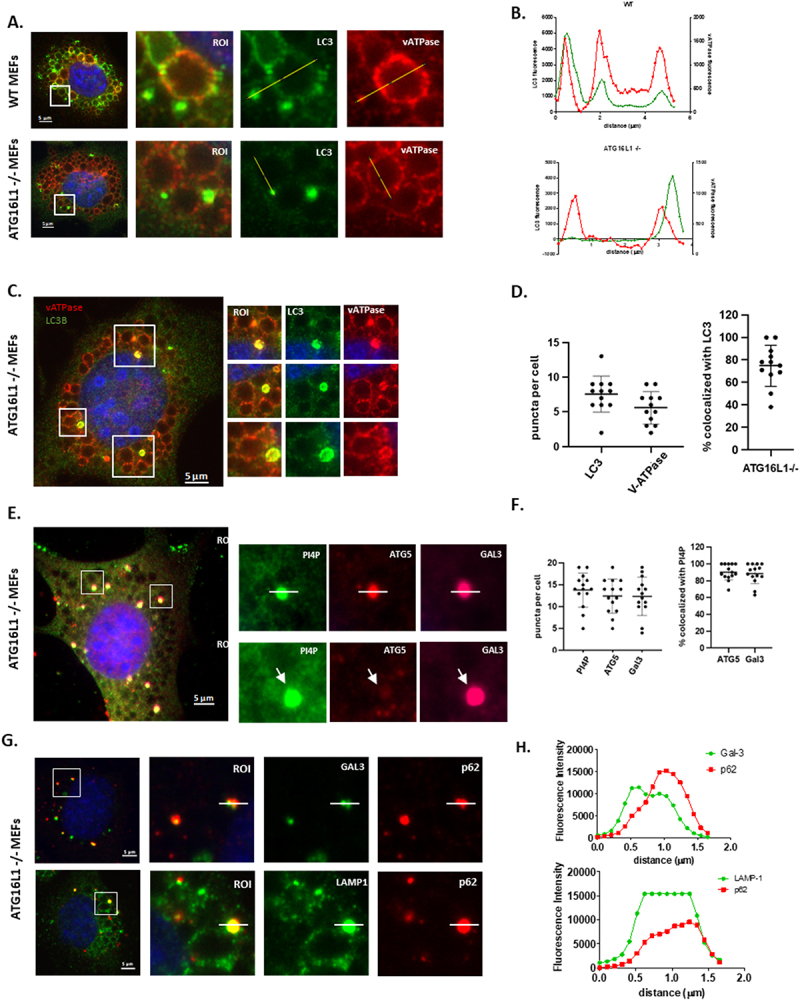
Conjugation of LC3 to ruptured lysosomes in the absence of ATG16L1. Control and ATG16L1-/- MEFs were incubated chloroquine (100 µM) for 2 hours as indicated, scale bar 5 um. Panel A. Immunostaining for LC3 (green) and V-ATPase (red). Panel B. distribution of LC3 (green and V-ATPase (red) assessed from line profiles. Panel C. Images of ATG16L1-/- MEFS where small puncta of LC3 co-localise with V-ATPase. Panel D. Co-localisation of LC3 and V-ATPase quantified by Imaris^TM.^ Panel E. Atg16L1-/- MEFs immunostained for PI4P (green), galectin-3 (red) and ATG5 (far red purple). Panel F. Co-localisation of PI4P (green), galectin-3 (red) and ATG5 (purple) quantified by Imaris^TM^. Panel G. immunostaining for p62 (red) and galectin-3 or LAMP1 as indicated. Panel H. Distribution of p62 (red) and galectin-3 or LAMP1 (green) assessed from line profiles.

The possibility that the small LC3 puncta were derived from lysosomes was tested by immunostaining for lysosome marker proteins. Microscopy and line profiles (Figure 5A,B) showed that following incubation of WT MEFs (Figure 5A top row) with chloroquine, LC3 colocalised with the membrane-spanning ATP6V1D subunit of the V-ATPase on the limiting membrane of large 3-4 μm diameter swollen lysosomes and was also present in the smaller bright LC3 puncta. In ATG16L1-/- MEFs (Figure 5A lower row, Figure 5B) the LC3 signal was absent from swollen lysosomes positive for V-ATPase but was again seen in small bright LC3 puncta close to lysosomes. Careful analysis of ATG16L1-/- cells (Figure 5C) showed that the LC3 signal was often located to a ring of fluorescence, the ring diameter of approx. 1.0 μm, was however less than that of the swollen lysosomes, but the presence of the V-ATPase in 80% of the LC3 puncta (Figure 5D), was consistent with the small puncta being derived from lysosome membranes. Evidence that the lysosome membranes in the puncta were permeabilised was provided by galectin-3 staining (Suppl. Figure 4). Regions of interest show that galectin-3 was located to a ring of fluorescence that colocalised with the V-ATPase at the perimeter of small puncta. The V-ATPase also located to large swollen lysosomes, but these were negative for galectin 3.

Lysosome permeabilization in response to LLOMe results in movement of phosphatidylinositol 4-kinase (PI4K2A) to the lysosome where it produces PI4P. Recruitment of oxysterol binding protein-related proteins (ORPs) 9-11 by PI4P promotes interactions with OSBP and VAPA/B to transport cholesterol and phosphatidylserine (PS) from the ER to the lysosome to facilitate lysosome repair [4]. In agreement with the results above, immunostaining following incubation of ATG16L1-/- MEFs with chloroquine (Figure 5E,F) showed that PI4P was recruited to small puncta containing galectin-3 indicating exposure of luminal galactosides. The majority (85%) of the PI4P/galectin-3 positive puncta also contained ATG5 (Figure 5F) suggesting recruitment of the ATG5-ATG12 complex required to conjugate LC3 to membranes. Further analysis showed that the galectin 3 puncta also contained LAMP1 and autophagy cargo receptor p62 (Figure 5G,H).

### Conjugation of LC3 to lysosomes in the absence of ATG16L1 requires TECPR1

**Figure 6.**
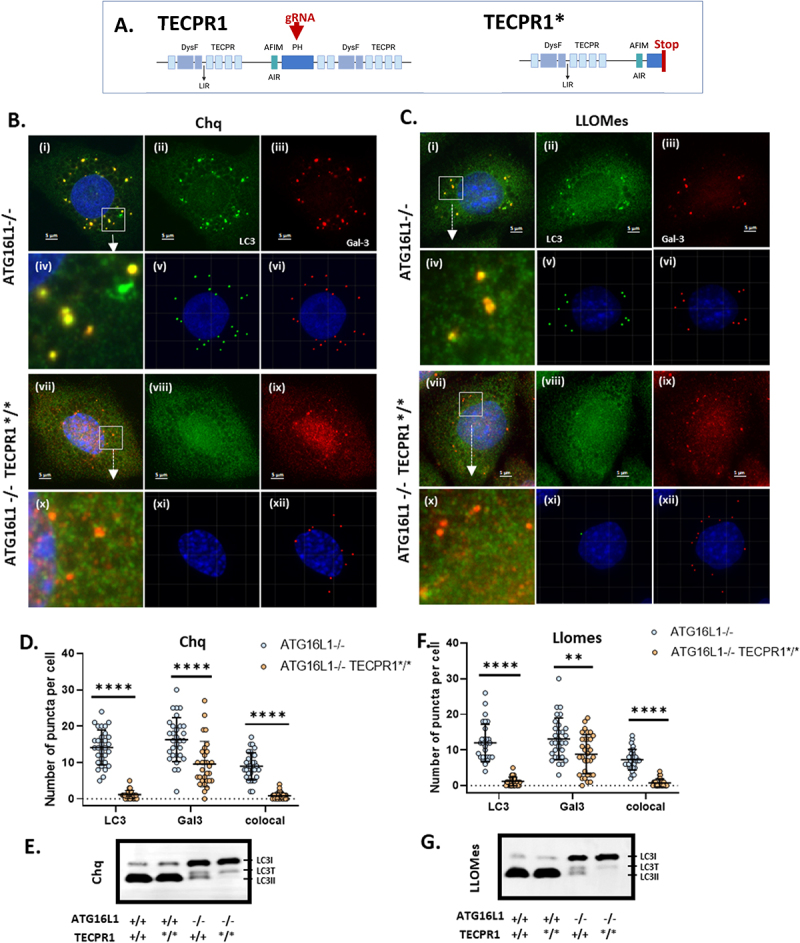
Conjugation of LC3 to damaged lysosomes in the absence of ATG16L1 requires TECPR1. Panel A. Domain map of TECPR-1 showing site of insertion of stop codon by custom CRISPR gRNA. ATG16L1-/- MEFs and gene edited ATG16L1-/- MEFS expressing truncated TECPR1* were incubated in nutrient media with chloroquine (100 µM, panel B) or LLOMes (1 mM panel C). Cells were fixed and immunostained for LC3(green) and galectin-3 (red). Rendered images (v and vi, xi and xii) were used to count puncta. Higher magnification images of boxed ROI are shown in iv and x. The graphs in panels D and F compare numbers of LC3 puncta co-localised with galectin-3 counted from rendered images of 30 cells incubated with chloroquine (panel D) or LLOMEs (panel F), P-values were calculated using multiple t-test (****P < 0.0001). Panels E and G show assessment of LC3II by western blot for LC3 for chloroquine (panel E) or LLOMes (panel G). Scale bar 5 μm.

Published pull down experiments and structural analysis have suggested that the TECPR1 binds ATG5 through an ATG5-interaction motif shared with ATG16L1 [29,30,32]. Recent work shows that the TECPR1:ATG5-ATG12 complex conjugates LC3 to membranes following physical damage to vacuoles [34,35] or vacuoles containing Salmonella [33]. The role played by TECPR1 in conjugation of LC3 to lysosomes in response to osmotic swelling induced by chloroquine, or following formation of pores by LLOMe polymers, was tested using gene editing to inhibit TECPR1 activity in the ATG16L1-/- MEFS. Guide RNAs were introduced by lentivirus transduction and sequencing of cell clones resistant to puromycin showed that guide RNAs generated a stop codon in exon 12 of the Tecpr1 gene (Suppl. Figure 5). This disrupted the central PH domain and prevented translation of the C-terminal dysferlin (DysF) domain that contributes to sphingomyelin binding [35] and the C-terminal tectonin β-propeller repeat (TECPR). This modification is labelled TECPR* to indicate truncation rather than complete removal (Figure 6A). Previous studies [30,31] have shown that TECPR1 co-localises with late endosomes and lysosomes. Immunostaining for TECPR1 in WT MEFS (Suppl. Figure 6A i-ii), and cells lacking ATG16L1 (Suppl. Figure 6B i-ii) shows that when cells were incubated in nutrient medium TECPR located to a crescent of immunostaining close to the nucleus, and the cells lacked damaged lysosomes indicated by lack of galectin 3 puncta. The crescent of immunostaining was less compact for the truncated TECPR1*. Following incubation of control MEFS with chloroquine (Suppl. Figure 6A), the unmodified endogenous TECPR1 was located to swollen LAMP1-positive vacuoles (Suppl. Figure 6A iii-iv) and to small galectin-3 positive puncta dispersed throughout the cell (Suppl. Figure 6A iii-vii). Interestingly, the TECPR signal on the lysosome membrane was not uniform and appeared to be located to domains of LAMP1 staining. In contrast in WT cells chloroquine failed to induce movement of the truncated TECPR* (Suppl. Figure 6B iii-vii) from the crescent of perinuclear immunostaining, and TECPR1* was absent from LAMP1 or galectin-3 positive structures (Suppl. Figure 6B iii-vii). Similar results were obtained for MEFs lacking ATG16L1 where chloroquine induced translocation of endogenous TECPR1 (Suppl. Figure 6C), but not the truncated TECPR1* (Suppl. Figure 6D), to LAMP1 (iv-v) or galectin 3 (v-vii) positive structures. This indicated that translocation of TECPR1 to did not require ATG16L1, and that the TECPR1* truncation was successful in preventing association of TECPR1 with both swollen lysosomes and ruptured lysosome membranes. The effect of the truncation on translocation of TECPR1* to ruptured galectin-3 positive lysosomes is shown in more detail in Suppl. Figure 7. Regions of interest on the right of the figure show that small puncta containing galectin 3 recruit endogenous TECPR1 (Suppl. Figure 7A) but do not recruit the truncated TECPR1* (Suppl. Figure 7B) which remained close to the nucleus. The subcellular location of endogenous TECPR1 has not been described in detail previously but the perinuclear distribution reported here suggests membrane compartments associated with the Golgi. Previous studies report the perinuclear distribution of EGFP-TECPR1 in resting cells but do not assign an organelle [35]. TECRP1-FLAG locates to small cytosolic puncta [30] which increase in number and co-stain for LC3 after induction of autophagy, and as reported here for endogenous TECPR1, the TECPR1-FLAG associates with ring-like lysosomes/autolysosomes in response to chloroquine. TECPR1 contains a central PH domain that binds PI4P [31] to target TECPR1 to lysosomes. The PH domain was disrupted during truncation of TECPR1* and this may contribute to the lack of translocation of TECPR1* to PIP4 positive lysosomes in response to chloroquine.

The effect of TECPR1 truncation on conjugation of LC3 to lysosomes in response to chloroquine in ATG16L1-/- cells is shown in Figure 6B,C. Bright puncta positive for LC3 and galectin-3 were generated in the ATG16L1-/- MEFS expressing endogenous TECPR1 (Figure 6B i-iii). High magnification (Figure 6B iv) and rendered images (Figure 6B v-vi) showed that the bright puncta were positive for both signals. Galectin-3 positive puncta were also formed in ATG16L1-/- MEFs expressing the truncated TECPR1* (Figure 6B vii-ix) but high magnification (Figure 6B x) and rendered images (Figure 6B xi-xii) showed that these galectin-3 positive puncta were negative for LC3. Quantitation of puncta (Figure 6D) showed an almost complete loss of LC3 puncta following truncation of TECPR1*. This was consistent with western blot analysis (Figure 6E) showing that chloroquine was unable to induce LC3II in ATG16L1-/- MEFS following truncation of TECPR1*. The experiment was repeated for cells incubated with LLOMe (Figure 6C). Again, bright galectin-3 puncta were produced in both cell types indicating damaged lysosomes, but damaged lysosomes did not recruit LC3 in ATG16L1-/- cells expressing the truncated TECPR1* (Figure 6C vii-xii). This was confirmed by quantitation of puncta in cells (Figure 6F) and western blot (Figure 6G). Interestingly, the numbers of galectin-3 puncta generated by chloroquine or LLOMe were reduced in ATG16L1-/- cells expressing the inactive truncated TECPR1* suggesting that TECPR-1 may facilitate access of galectin-3 to the luminal sugars of the lysosomes. This is consistent with observations of Boyle et al [33] who show that silencing of TECPR1 reduces recruitment of galectin-8 to vacuoles damaged by Salmonella resulting in slower entry of bacteria into the cytosol.

**Figure 7.**
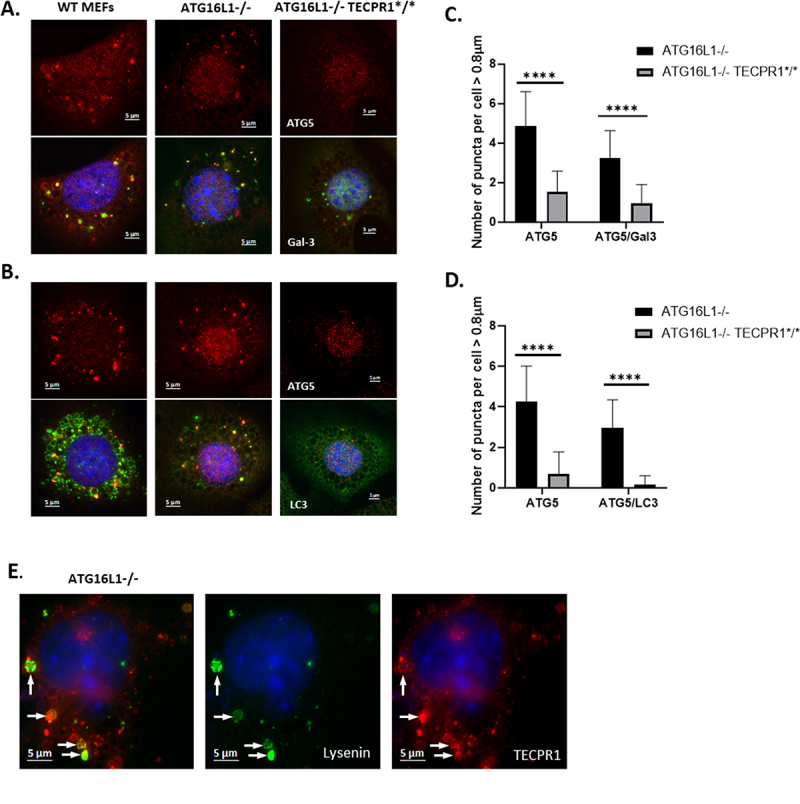
TECPR-1 facilitates recruitment of LC3 and ATG5 to damaged lysosomes in the absence of ATG16L1. Control (WT), Atg16L1-/- and ATG16L1-/- MEFS expressing truncated TECPR1* were incubated for 2 hours in nutrient media containing chloroquine (100 µM). Panel A. Cells were fixed and immunostained for ATG5 (red) and galectin-3 (green). Panel B. Cells were fixed and immunostained for ATG5 (red) and LC3 (green). Panel C. Quantification and composition of puncta positive for ATG5 and galectin 3. Panel D. Quantification and composition of puncta positive for ATG5 and LC3. n ≥ 30 cells were quantified by Imaris, P-values were calculated using multiple t-test (****P < 0.0001). Scale bar 5 μm. Panel E. ATG16L1-/- MEFS expressing GFP-lysenin (green) and TECPR1-RFP (red) were incubated in nutrient media containing chloroquine (200 µM) for 30 minutes. Scale bar 5 μm.

TECPR1 contains an ATG5-interaction motif shared with ATG16L1. The ability of TECPR1 to recruit ATG5-ATG12 onto lysosomes in response to chloroquine was tested by immunostaining for ATG5. Puncta positive for both galectin-3 and ATG5 were produced in WT MEFS and in MEFS lacking ATG16L1 (Figure 7), but ATG5 recruitment was greatly reduced in Atg16L1-/- MEFS expressing the truncated TECPR1* (Figure 7A). There was a similar reduction in recruitment of ATG5 to bright puncta positive for LC3 (Figure 7B) in cells expressing TECPR1*. Counts of small bright puncta (Figure 7C,7D) confirmed that recruitment of ATG5 to puncta positive for galectin-3 or LC3 in ATG16L1-/- MEFs was greatly reduced following truncation of TECPR1*.

### Recruitment of galectin-3 and GFP-lysenin to lysosomes correlates with release of dextran

**Figure 8.**
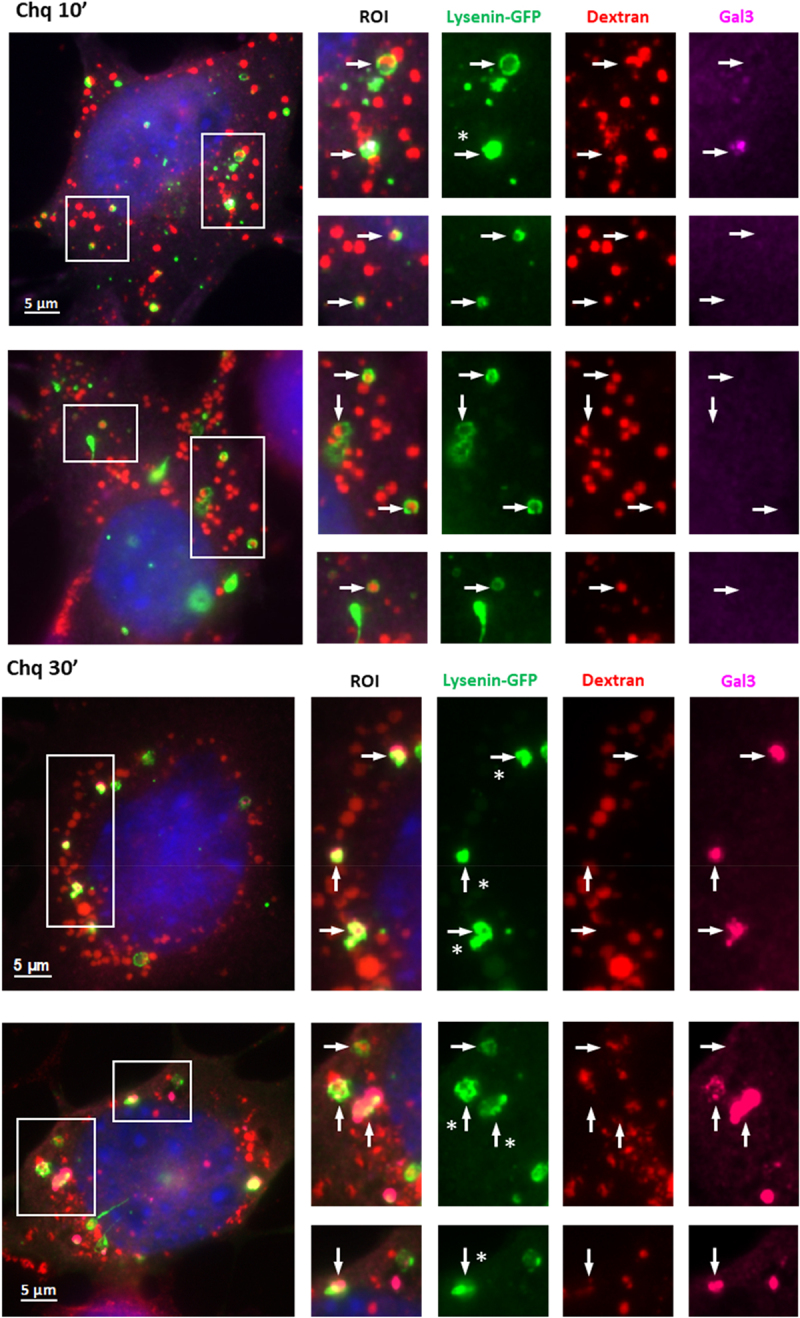
Recruitment of galectin-3 and GFP-lysenin to lysosomes correlates with release of dextran. ATG16L1-/- MEFS expressing GFP-lysenin were cultured in Dextran-Red^R^ (300 mg/ml) to label lysosomes and incubated with chloroquine (200 µM) for 10 or 30 minutes as indicated. Images show distribution of GFP-lysenin, Dextran-Red^R^ and galectin-3 (far red). Asterisks indicate puncta positive for lysenin and galectin-3 that are negative for dextran.

The relationship between sphingomyelin exposure and TECPR1 was examined using ATG16L1-/- MEFs co-expressing TECPR1-RFP and the sphingomyelin probe lysenin-GFP [36]. Figure 7E shows that chloroquine generated swollen vacuoles positive for both lysenin-GFP and TECPR1 indicating recruitment of TECPR1 onto vacuoles that have exposed sphingomyelin in response to osmotic stress. In the next experiment the lysenin-GFP probe was expressed in ATG16L1-/- MEFS cultured with Dextran-Red^R^ to label lysosome content. Representative images recorded 10 and 30 minutes after addition of chloroquine are shown in Figure 8 and images at 15 and 40 minutes are presented in Supplemental figure 8. In each case lysenin-GFP was located to swollen vacuoles and regions of interest show that vacuoles positive for lysenin-GFP could be negative (arrows) or positive (arrows with asterisk) for galectin-3. The dextran was retained in lysenin positive vacuoles that lacked galectin-3 but was lost from vacuoles positive for galectin-3. The results suggested that vacuoles positive for galectin 3 (arrows with asterisk) generated in response to chloroquine had lost their luminal content of dextran following rupture leading to exposure of glycans.

**Figure 9.**
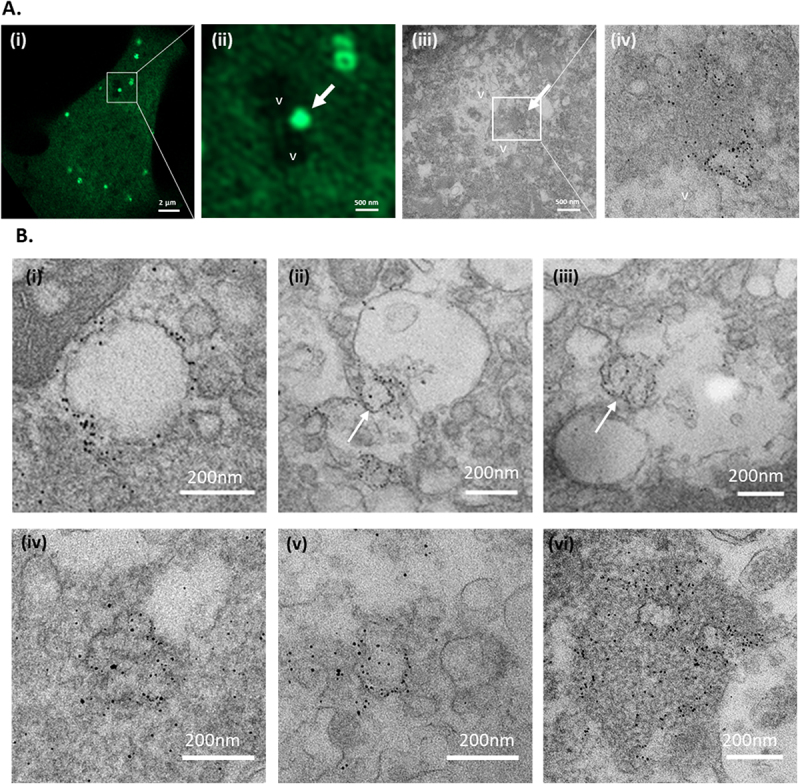
Ultrastructure of LC3 puncta analsyed by CLEM. MEFs growing on grid dishes were transduced with LC3-GFP adenovirus, incubated with chloroquine (100 µM) and observed by live cell imaging. Fixed cells permeabilised with saponin were incubated with rabbit antibody against GFP followed by antirabbit nanogold and silver enhancement. Panel A. ATG16L1-/- MEFs of interest were identified by correlating the grid and cell pattern with previously acquired brightfield images. Panel B gallery of images generated from WT (i-iii) and ATG16L1-/- MEFs (iv-vi).

The ultrastructure of the small bright LC3 puncta was analysed in MEFs transduced by an adenovirus expressing GFP-LC3 (Figure 9). CLEM was used to locate fluorescence signals close to vacuoles swollen by chloroquine that correlated with strong immunogold labelling for GFP (Figure 9A). Bright fluorescent puncta close to dark vacuoles (arrows in Figure 9A i and ii) were used to locate equivalent structures in the electron micrographs (Figure 9A. iii and iv). Electron micrographs at higher magnification (Figure 9A vi-viii) show that the fluorescent puncta could be superimposed on packed poorly defined membranes staining strongly with immunogold directed at GFP. Further images compare WT and ATG16L1/-/ cells (Figure 9B and Suppl. Figure 9). In WT cells the GFP-LC3 was largely localised to the limiting membrane of vacuoles (Figure 9B i-iii) and to smaller electron dense (100-500 nm) structures containing poorly defined membranous material. In ATG16L1-/- MEFs the strong immunogold labelling was absent from the membrane of the vacuoles but concentrated in small electron dense structures containing packed membranes (Figure 9B iv-vi).

**Figure 10.**
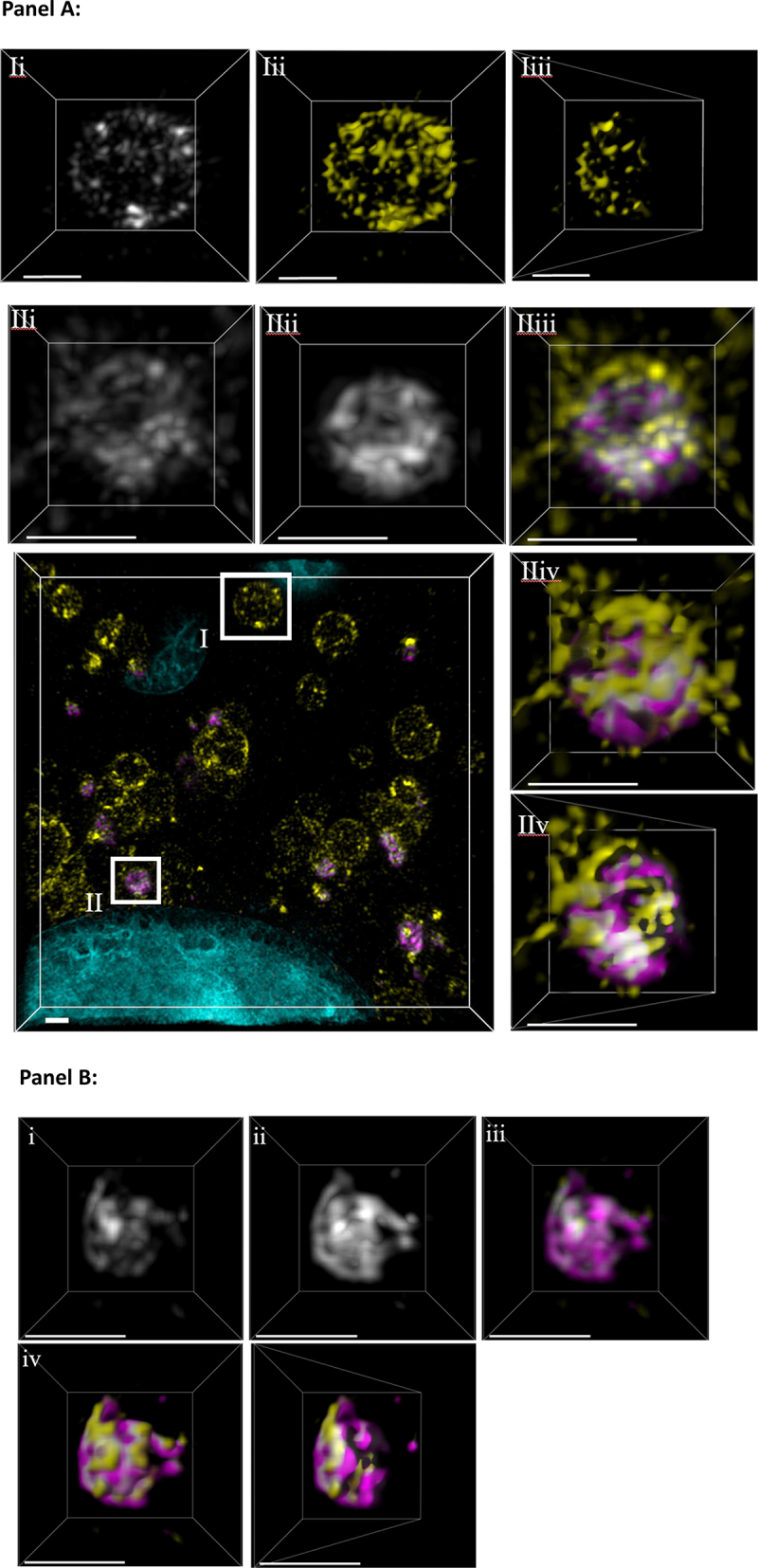
Analysis of LC3 and galectin-3 distribution on damaged lysosomes by structured illumination microscopy: MEFs were incubated nutrient media containing chloroquine (100 µM) for 2 hours and then stained with DAPI (cyan) and immunostained for LC3 (yellow) and galectin3 (magenta) and imaged by 3D-SIM. Panel A: control MEFS. A swollen vacuole (I) recruits LC3 (I-i, maximum intensity projection-MIP) but not galactin3. To visualise this better, using Imaris, a 3D rendered surface was applied (I-ii) and then bisected (I-iii) showing the hollow vacuole interior. Analysis of small dense puncta (II) shows that LC3 (II-i, yellow MIP) and galectin3 (II-ii, magenta MIP) are recruited together as seen in the coloured overlay (II-iii). A 3D rendered surface (II-iv) bisected (II-v) shows the densely packed interior of this puncta. Scale bar = 1 µm. Panel B: ATG16L1-/- MEFs. Analysis of small dense puncta shows that LC3 (i, yellow MIP) and galectin3 (ii, magenta MIP) are recruited together as seen in the coloured overlay (iii). A 3D rendered surface (iv) bisected (v) shows the densely packed interior of the puncta. Scale bar = 1 µm.

The nature of the packed membranes was analysed further using structured illumination microscopy to compare swollen lysosomes with small LC3 puncta containing galectin-3 (Figure 10). Rendered 3D visualisations of swollen vacuoles formed in control MEFS (Figure 10A AIi-iii and Suppl. movie 1) recruited LC3 but were negative for galectin-3. Maximum intensity projections (MIP) and 3D surface rendering show that the vacuole lacked galectin 3 and that the LC3 signal was absent from the interior of the vacuole. Analysis of bright puncta formed in control MEFs (Figure 10A IIi-v and Suppl. movie 2) showed that the puncta contained signals for LC3 and galectin-3 that were interwoven throughout the puncta. Bisected images taken from MIPs (Figure 10A IIiv-v) showed that the interior of the structure was packed with membranes containing both proteins. Similar analysis of bright puncta formed in ATG16L1-/- cells (Figure 10B, Suppl. Movie 3) again showed that LC3 and galectin signals were interwoven throughout the puncta. These images are consistent with the detection of packed membranes labelling strongly for LC3 by CLEM (Figure 9) and images and line profiles presented in Figure 1. Recent studies [11] show that lysosome damage by LLOMes results in release of lysosome membrane proteins such as LAMP2 into the cytosol as small lysosomal membrane complexes that become a substrate for autophagy. The small diameter of those structures and their content of LAMP2 resemble the electron dense structures reported here. This suggests that lysosomal membrane complexes released from ruptured lysosomes may be a target for TECPR1 mediated conjugation of LC3.

### Role played by the LIR motif and PH domain of TECPR1 during membrane association and conjugation of LC3 to damaged lysosomes

**Figure 11.**
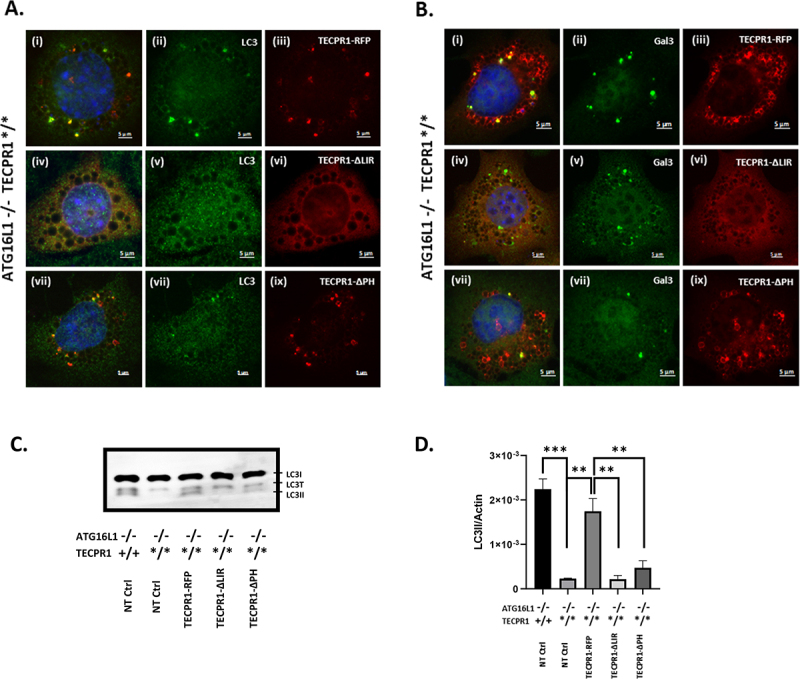
Role played by the LIR motif, PH domain of TECPR-1 during conjugation of LC3 to damaged lysosomes. Gene edited ATG16L1-/- MEFS expressing truncated TECPR1* were transfected with plasmids expressing full length TECPR-1-RFP (i-iii), TECPR-1 lacking the LIR within the N-terminal TECPR domain (TECPR1-∆LIR-GFP pseudo red, iv-vi) or TECPR-1 lacking the central PH domain (TECPR1-∆PH-GFP pseudo red, vii-ix). Cells were incubated with chloroquine (100 µM) for 2 hours. Panel A shows counter staining for LC3 (green) and panel B counter staining for galectin 3 (green). Panel C and D. Western blot analysis and relative quantification of LC3II. ATG16L1-/- TECPR*/*MEFS transfected with indicated plasmids were incubated in nutrient media containing chloroquine (100 µM) for 2 hours. Data represent mean ± S.E. of three independent experiments (***P < 0.001). Scale bar 5 μm.

TECPR1 uses a central PH domain and an N-terminal LIR domain to tether lysosomes to autophagosomes [31]. The role played by the LIR of TECPR1 in recruiting LC3 to galectin-3 positive damaged lysosomes was tested by reconstituting ATG16L1-/- cells expressing the inactive truncated TECPR-1* with full length TECPR1-RFP, or a mutant protein (TECPR1-∆LIR) lacking residues W175-178 in the LIR required for LC3 binding (Figure 11). Expression of full length TECPR1 reconstituted formation of LC3 puncta in response to chloroquine and the puncta were positive for TECPR1-RFP (Figure 11A i-iii) and galectin-3 (Figure 11B i-iii). Conjugation of LC3 to lipids was confirmed by the appearance of LC3II on western blot (Figure 11 C&D). This confirmed that TECPR1 could conjugate LC3 independently of ATG16L1. Expression of the TECPR1-∆LIR mutant failed to restore LC3 puncta (Figure 11A iv-vi) and TECPR1-∆LIR was absent from lysosomes positive for galectin-3 (Figure 11B iv-vi) and LC3II was absent from western blots (Figure 11C,D). The TECPR1-∆LIR mutant was not recruited to galectin-3 puncta (Figure 11B iv-vi). Given that the W175-178 residues lie within the TR1 domain required for lysosome binding it is possible that the W175-178 mutation inactivates both the LIR and lysosome binding properties of the TR1 domain making recruitment of TECPR1 to damaged lysosomes no longer possible. Similarly, the W175-178 mutation may have disrupted the sphingomyelin binding domain in residues of the N-terminal Dys-F domain (residues 63 to 170) required for binding to sphingomyelin exposed early on stressed lysosomes [34,35]. Damaged lysosomes generated by chloroquine or LLOMe were enriched for PI4P. The role played by the PI4P-specific PH domain of TECPR1 in recruiting LC3 to damaged lysosomes was tested using TECPR1 lacking the PH domain (TECPR1-∆PH). A small number of LC3 puncta were restored by TECPR1-∆PH RFP compared to unmodified TECPR1 (Figure 11A vi-ix) and these puncta co-located with TECPR1-∆PH RFP, but numbers of LC3 puncta were lower than seen for full length TECPR1. The TECPR1-∆PH RFP mutant located to puncta, and some co-stained for galectin-3 (Figure 11B vi-ix) while others were negative for galectin-3 or LC3. Reduced LC3 conjugation by TECPR1-∆PH RFP was also evident in western blots showing that levels of LC3II generated in response to chloroquine were 10-20% of those generated by full length TECPR1 (Figure 11C,D). This suggested that recruitment of LC3 to ruptured lysosomes was greatly reduced following loss of the PH domain, but not lost completely. It is possible that residual binding to lysosomes is maintained by the TRI and DysF domains that remain intact in the TECPR1-∆PH mutant.

## Discussion

**Figure 12.**
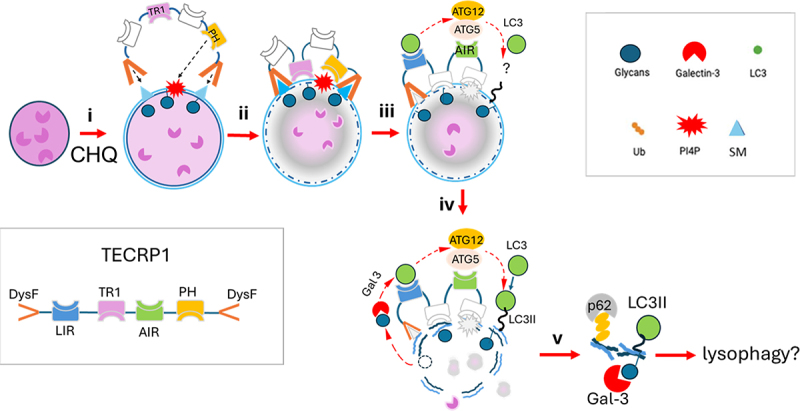
TECPR1-dependent conjugation of LC3 to lysosomes in response to osmotic imbalance. i). Dysferlin domains of TECPR1 bind sphingomyelin exposed early during osmotic stress. ii). TECPR1 binding is enhanced by TR1 dependent tethering to the lysosome and binding of the PH domain to PI4P. iii). The AIR and LIR domains of TECPR1 recruit the ATG5-ATG12 conjugate and LC3, but conjugation of LC3 is inefficient. iv). Lysosome rupture exposes luminal galactose residues to recruit galectin-3 to facilitate conjugation of LC3 by the TECPR1:ATG5-ATG12 complex. v). Ruptured lysosome membranes containing LC3II, p62 and galectin 3 may be removed by lysophagy.

This study shows that a TECPR1:ATG5-ATG12 complex provides the E3-ligase-like activity necessary for conjugation of LC3 to endo-lysosome compartments in response to osmotic imbalance induced by chloroquine or membrane damage induced by LLOMe. By analogy with ATG16L1, it is possible that ATG5 in the TECRP1:ATG5-ATG12 complex induces a conformational change in ATG3 to transfer the C-terminal carboxyl group of LC3 from a thioester bond to amino groups exposed by PE or PS. The role played by TECPR1 during conjugation of LC3 to lysosomes is summarised in Figure 12. Recruitment of TECPR1 to vacuole membranes is mediated by dysferlin (DysF) domains that bind sphingomyelin exposed at early stages of lysosome stress before exposure of galactosides to galectin-3 [33–36]. The N and C-terminal dysferlin domains of TECPR1 bind sphingomyelin (Figure 12 i) and TECPR1 binding is enhanced by TR1-dependent tethering to the lysosome and binding of the PH domain to PI4P (Figure 12 ii and ref [31]). The ATG5 interacting region (AIR) and LIR domains of TECPR1 recruit the ATG5-ATG12 conjugate and LC3, but conjugation of LC3 to intact swollen lysosomes is inefficient (Figure 12 iii) and may require additional membrane damage signals associated with membrane rupture such as galectin-dependent autophagy pathways upstream of the E3 ligase activity of ATG5-ATG12 (Figure 12 iv and refs [7–9]).

LC3 was located to small puncta of approx. 1.0 μm diameter, rather than to the limiting membrane of the swollen lysosomes. The puncta contained lysosome marker proteins such as the ATP6V1D stalk subunit of the V-ATPase and LAMP1 and the presence of galectin-3 and PI4P provided evidence for lysosome damage. In studies of bacteria-containing vacuoles [33] a lysenin probe indicating sphingomyelin exposure generates continuous uniform labelling of vacuoles early during infection suggesting intact membranes, but the signal breaks down during recruitment of galectin-8. At the same time large gaps of 100-200 nm diameter appear in the vacuole membrane suggesting that galectin-8 enters through large pores prior to exit of bacteria into the cytosol [33]. Lysenin-positive lysosomes, indicating exposure of sphingomyelin, were observed soon after addition of chloroquine, and many galectin-positive vacuoles, indicating the presence of large pores, had lost their luminal content of dextran, while galectin-negative vacuoles retained dextran. This makes it possible that the small LC3/galectin 3 positive puncta observed in this study result from lysosome membrane rupture, loss of content/osmotic pressure and vacuole collapse. This was consistent with ultrastructural analysis of LC3 positive membranes showing that LC3 and galectin-3 were recruited to dense structures packed with membranes rather than membrane vacuoles.

In common with other ATG8ylation pathways such as autophagy and V-ATPase-activated CASM, the direct conjugation of LC3 to ruptured lysosome membranes by TECPR1 has the potential to make membranes substrates for degradation by healthy lysosomes [41,42]. LC3 provides a platform for interactions with SNARE proteins (STX17, SNAP29, VAMP8) and tethering proteins (HOPS, PLEKHM1) to promote lysosome fusion [13,14]. TECPR1 also tethers lysosomes to autophagosomes [31] and tethering of ruptured lysosomes may be enhanced by the central PH domain in TECPR1 that binds lysosomes in response to the production of PI4P during lysosome damage. Understanding how membrane remnants are taken up into lysosomes will require further work. Tethering by TECPR1 may facilitate fusion, as has been described for the removal of autophagosomes containing protein aggregates [30,31]. Alternatively, membrane remnants may be taken into lysosomes by microautophagy/microlysophagy. Conjugation of LC3 to damaged lysosomes by TECPR1 has also been shown to facilitate lysosome repair [34]. Recent LC3 interactome analysis has shown that lysosome damage induces binding of LC3 to lysosome membrane protein LAMP2 [11]. LC3 also recruits TBC1D15, a Rab7 GTPase activating protein that activates Rab7 to promote dynamim-2 dependent release of proto-lysosomes making their lipids available for lysosome repair. The lysosome damage response also activates TFEB to promote transcription of genes important during autophagy and lysosome biogenesis (15). It will be interesting to determine if TECPR1-mediated conjugation of LC3 to damaged lysosomes also contributes to TFEB activation in response to damage.

The experiments reported here have taken advantage of ATG16L1-/- cells to reveal TECPR1:ATG5-ATG12-dependent conjugation of LC3 to damaged lysosome membranes. These events may be masked in wild type cells if damaged membranes are removed by ATG16L1-dependent canonical autophagy. This means that the overall contribution made by the TECPR1:ATG5-ATG12 pathway in wild type cells where alternative autophagy pathways driven by ATG16L1 are active, remain to be determined. Comparisons of LC3 lipidation between wild type and ATG16L1-/- cell lines in response to LLOMe [34] suggest TECPR1 dependent conjugation accounts for between 7 and 25% of total LC3 lipidation. ATG16L1 and TECPR1 form mutually exclusive complexes with ATG5-ATG12 [30] raising the possibility that competition for binding to ATG5-ATG12 can further affect the relative roles played the two complexes in wild type cells. It is interesting to speculate that ATG8ylation by TECPR1 may have roles in addition to responding to vacuole damage. TECPR1 can conjugate ATG8 family proteins such as GABARAP to membranes [35] and TECPR1 is recruited to depolarised mitochondria [29] and MEFs from TECPR1-/- mice show defects in removal of protein aggregates and disposal of depolarised mitochondria implicating roles for TECPR1 in aggregate clearance and mitophagy [29].

### Data management

Data is generated and stored at a central imaging facility and is available upon request. The raw data were generated at [Henry Wellcome laboratory for cell imaging, University of East Anglia, Norwich, NR4 7TJ. Derived data supporting the findings of this study are available from the corresponding author [TW] on request. Microscopy imaging data (both the raw images and the analysed images) and associated metadata, will be also be made publicly available through the OMERO platform, with the files stored on internal servers held at the Quadram Institute. The DOI for this data set will be submitted with the acceptance of the publication for publishing.

Raw image data and associated metadata referenced in the publication will be submitted to the BioImage Archive https://www.ebi.ac.uk/bioimage-archive/ where it will be given an ascension number. This archive is hosted by the EMBL European Bioinfomatics Institute. Submission to the BioImage Archive requires an accepted peer-reviewed paper that corresponds to the image data set, therefore an ascension number will be generated upon the acceptance of the paper and submitted before publication.

### Conflict of interest

The authors have declared that no conflict of interest exists.

### Author contributions

TW designed the research. YW, MJ, MW, KK, MR, GK, performed cell culture experiments, YW and JM carried out live cell imaging. TW, YW and PV designed and carried out CLEM experiments. JL and CW designed and performed SIM analysis. TW wrote the manuscript. UM, ML provided essential reagents and knowledge and YW, ML and UL proofread and edited the manuscript.

## Methods

### Antibodies and reagents

rabbit-LC3A/B (CST #4108), mouse-LC3B (CST #83506), rat-Lamp1 (abcam [1D4B] ab25245), rat-galectin3 (Santa Cruz M3/38 sc-23938), rabbit-Atg5 (GTX113309), GP-p62 (Progen GP62-C), mouse-EEA1 (MBL M176-3), mouse-Actin (sigma A2228), anti-TECPR1 (Novusbio NBP1-84206) ATP6V1D (Abcam ab157458). Chloroquine (Chq) (Sigma, C-6628, 100 µM), monensin (Mon) (Sigma, M-5273, 100 µM), Leu-Leu methyl ester hydrobromide (LLomes) (Sigma, L-7393, 1 mM), bafilomycin A (Baf) (Sigma, B-1793, 100 nM), diphenyleneiodonium chloride (DPI) (Sigma, D-2926, 10 µM), phloretin (phl) (Sigma, P-7912, 180 µM), wortmannin (Wtm) (Sigma, W-1628, 100 nM)

### Plasmid and transfection

Cells were transfected with FuGENE® HD Transfection kit according to the manufacturer’s instructions, harvested or analysed 24–48 hours post transfection. Tecpr1-RFP, Tecpr1-W175A/I178A-GFP, Tecpr1-dPH-GFP were generous gifts from Stephane Blanchard and Thomas Wollert and described in Wetzel et al 2020.

### Cells and cell culture

Mouse embryonic fibroblasts (MEFs) were cultured in DMEM (ThermoFisher scientific, 11,570,586) with 10% foetal bovine serum and 2 mM L-glutamine and 100 U/ml penicillin/streptomycin and incubated in 5% CO2 at 37°C. Cells were starved by incubating in Hank’s balanced salt solution (HBSS; Invitrogen, 14025-076) which lacks amino acids for the specified length of time to stimulate autophagy.

### Truncation of Tecpr1 in MEFS

Gene editing of Tecpr1 in MEFs was achieved using custom CRISPR gRNA lentivirus transduction particles (Mission, Sigma Aldrich). MEFs were infected with the CRISPR gRNA lentiviruses in Opti-Minimal Essential Medium (Opti-MEM) at MOI of 1, supplemented by 16 μg/mL hexadimethrine bromide. Transduced cells were selected by 10 μg/mL puromycin and single cell colonies were isolated by dilution. Genomic regions containing the editing site were amplified by PCR and screened for frameshift mutations by sequencing. (gRNAs 5’-AGCAGTTCACAGGGCACGA and gRNA 5’- TGTACACGGGCGGCTATGG)

### Western blotting

Protein was extracted using M-PER (ThermoFisher Scientific, 78,501) with complete protease inhibitor cocktail (Sigma, 04693159001) for 30 min on ice. 30 μg protein from clarified samples was separated on a precast 4–12% gradient or 12% np gradient SDS-PAGE gel (Expedeon, NBT41212) and transferred to immobilon PVDF (Millipore, IPFL00010) for blotting. Membranes were probed using antibodies for ATG16L1 (MBL M150-3) and ACTB/actin (Sigma, A5441). Primary antibodies were detected using IRDye labelled secondary antibodies (LI-COR biosciences, 926–32,211, 926–68,020) at 1:10,000 dilution. Proteins were visualised using the Odyssey infrared system (LI-COR).

### Fluorescence microscopy

Cells grown on glass coverslips in 24-well plates were fixed at −20°C in ice cold methanol for 7 min, then blocked in 5% goat serum, 0.3% Triton X-100 in PBS (Sigma, G9023; X100) for 30 min. Cells were incubated with primary antibody overnight. Washed cells were incubated with secondary antibody anti-Rabbit IgG Alexa Fluor® 488 (Life Technologies, A11008), anti-Rat IgG Alexa Fluor®594, anti-Rat IgG Alexa Fluor®488 and anti-mouse IgG Alexa Fluor® 594, 2 h at room temperature, followed by counterstain with 4ʹ, 6 diamidino-2-phenylindole (DAPI; ThermoFisher Scientific, 10,116,287) and mounted on slides with Fluoromount-G from Southern biotech (ThermoFisher Scientific, 15,586,276). Cells were imaged on a Zeiss Imager M2 Apotome microscope with a 63X, 1.4 NA oil-immersion objective using 365 ± 40 nm excitation and 445 ± 25 nm emission for DAPI, 470 ± 20 nm excitation and 525 ± 25 nm emission for LC3.

### Correlative light electron microscopy

ATG16l1-/- MEFs were cultured on 35 mm Glass Bottom grid dishes (No. P35G-1.5-14-C-GRID; MatTek Corporation) and transduced with LC3-GFP by adenovirus. Live cell imaging was performed after incubation with chloroquine for 1 or 2 h, At the end point of live cell imaging, cells were fixed in 4% paraformaldehyde in PBS for 20 min and stored in 2% PFA in PBS until being quenched in 0.3 M glycine in PBS for 10 min, blocked in 0.1% BSAc (Aurion) in PBS for 30 min and permeabilized by incubation with 0.1% saponin and 0.1% BSAc in PBS. Samples were incubated with the primary antibody, anti-Rabbit-GFP, in for 1 h, washed and followed by secondary antibody, anti-Rabbit-nanogold (lot 18C070) for 30 min. Following washes with incubation buffers and distilled water, the samples were then sliver enhanced using the Aurion SE-EM kit for 30 min. After three washes in water, the samples were post-fixed for 20 min with 1% Osmium tetroxide in water, incubated with 3% uranyl acetate in water for 30 min, washed three times in water, dehydrated through an ascending series of ethanol, and embedded in Epon and polymerisation overnight at 60 °C. The coverslips were removed from the resin blocks by plunging briefly into liquid nitrogen and hot water. The cells of interest were identified by correlating the grid and cell pattern on the surface of the block with previously acquired brightfield images. The area of interest was trimmed into a small trapezoid block. Serial ultrathin sections of 70 nm thickness were cut on Leica UC7 ultramicrotome using a diamond knife and collected on carbon-coated mesh grids [43].

### Super-resolution three-dimensional structured illumination microscopy (3D-SIM)

Cells grown on No. 1.5 13 mm glass coverslips in 24-well tissue culture treated plates were treated with chloroquine for 2 h and then fixed at −20 °C in ice cold methanol for 7 min. Fixed cells were blocked in 5% goat serum and 0.3% Triton X-100 in PBS (Sigma, G9023; X100) for 1 h. Cells were incubated overnight with the antibodies mouse anti-LC3B antibody (CST #83506), rat anti-Lamp1 (Abcam [1D4B] ab25245), and/or, rat anti-galectin3 (Santa Cruz M3/38 sc-23938). Cells were washed and incubated with secondary antibody anti-Rat IgG Alexa Fluor®594 and/or anti-mouse IgG Alexa Fluor® 488 (Life Technologies), for 2 hr at room temperature, followed by counterstain with 4ʹ, 6 diamidino-2-phenylindole (DAPI; ThermoFisher Scientific, 10,116,287). Coverslips were then washed and mounted on slides with ProLong™ Diamond Antifade Mountant (Invitrogen P36965).

Super-Resolution 3D-SIM images were taken on the DeltaVision OMX Flex Microscope System (Cytiva, WA) using an Olympus 60x/1.42 NA PlanApochromat PSF oil objective and refractive index matched oil. 0.125 µm Z stacks spanning the focal range were captured using blue, green and red channels. 3D-SIM reconstructions were performed using SoftWorx 7.2.2 software (Cytiva) with the Weiner filters set to 0.03, 0.01 and 0.01 respectively. Imaged were aligned and corrected for chromatic aberration using 0.1 µm TetraSpeck™ microspheres (Invitrogen™, ThermoFisher Scientific, MA) beads in ProLong Diamond Antifade mountant. Reconstructed and aligned images were visualised in Imaris 10.0.1 having been converted using Imaris File Converter 9.8.2 (Oxford Instruments, Switzerland). Supplemental movies were made using Imaris 9.8.2. Images show the maximum intensity projection and 3D surface rendering with shading. Images have only been adjusted for brightness and contrast. The blue channel was used to identify cells and then removed from the visualisations.

### Statistics

Data were analysed using the Prism package (version 5.04 Graphpad Software). *P* values were set at 95% confidence interval. The statistical significance of difference in mean values was calculated by unpaired, two-tailed Student t test or one-way ANOVA. The statistical significance of difference of the percentages between two groups was calculated by Fisher’s exact test. P values less than 0.05 were considered statistically significant. No exclusion criteria were applied to exclude samples from analysis. All differences not specifically stated to be significant were not significant (p > 0.05). For all figures, *p < 0.05, **p < 0.01, ***p < 0.001, ****p < 0.0001.

## Supplementary Material

Supplemental Material

## References

[1] Aits S, Jäättelä M. Lysosomal cell death at a glance. J Cell Sci. 2013;126(9):1905–32. 23720375 10.1242/jcs.091181

[2] Radulovic M, Schink KO, Wenzel EM, et al. ESCRT-mediated lysosome repair precedes lysophagy and promotes cell survival. Embo J. 2018;37:37e99753. 10.15252/embj.201899753 PMC6213280 30314966

[3] Skowyra ML, Schlesinger PH, Naismith TV, et al. Triggered recruitment of ESCRT machinery promotes endolysosomal repair. Science. 2018;360(6384). doi:10.1126/science.aar5078 PMC6195421 29622626

[4] Tan JX, Finkel T. A phosphoinositide signalling pathway mediates rapid lysosomal repair. Nature. 2022;609(7928):815–821. 36071159 10.1038/s41586-022-05164-4 PMC9450835

[5] Bussi C, Mangiarotti A, Vanhille-Campos C, et al. Stress granules plug and stabilize damaged endolysosomal membranes. Nature. 2023;623(7989):1062–1069. 37968398 10.1038/s41586-023-06726-w PMC10686833

[6] Thurston TLM, Wandel MP, von Muhlinen N, et al. Galectin 8 targets damaged vesicles for autophagy to defend cells against bacterial invasion. Nature. 2012;482(7385):414–418. 22246324 10.1038/nature10744 PMC3343631

[7] Jia J, Abudu YP, Claude-Taupin A, et al. Galectins control mTOR in response to endomembrane damage. Mol Cell. 2018;70(1):120–135.e8. 29625033 10.1016/j.molcel.2018.03.009 PMC5911935

[8] Jia J, Claude-Taupin A, Gu Y, et al. Galectin-3 coordinates a cellular system for lysosomal repair and removal. Dev Cell. 2020;52(1):69–87.e8. 31813797 10.1016/j.devcel.2019.10.025 PMC6997950

[9] Chauhan S, Kumar S, Jain A, et al. TRIMs and galectins globally cooperate and TRIM16 and galectin-3 co-direct autophagy in endomembrane damage homeostasis. Dev Cell. 2016 Oct 10;39(1):13–27. 27693506 10.1016/j.devcel.2016.08.003 PMC5104201

[10] Bhattacharya A, Mukherjee R, Kuncha SK, et al. A lysosome membrane regeneration pathway depends on TBC1D15 and autophagic lysosomal reformation proteins. Nat Cell Biol. 2023;25(5):685–698. 37024685 10.1038/s41556-023-01125-9

[11] Eriksson I, Wäster P, Öllinger K. Restoration of lysosomal function after damage is accompanied by recycling of lysosomal membrane proteins. Cell Death Dis. 2020;11(5):370. 32409651 10.1038/s41419-020-2527-8 PMC7224388

[12] Maejima I, Takahashi A, Omori H, et al. Autophagy sequesters damaged lysosomes to control lysosomal biogenesis and kidney injury. Embo J. 2013;32(17):2336–2347. 23921551 10.1038/emboj.2013.171 PMC3770333

[13] McEwan DG, Popovic D, Gubas A, et al. PLEKHM1 regulates autophagosome-lysosome fusion through HOPS complex and LC3/GABARAP proteins. Mol Cell. 2015;57(1):39–54. 25498145 10.1016/j.molcel.2014.11.006

[14] Itakura E, Kishi-Itakura C, Mizushima N. The hairpin-type tail-anchored SNARE syntaxin 17 targets to autophagosomes for fusion with endosomes/lysosomes. Cell. 2012;151(6):1256–1269. 23217709 10.1016/j.cell.2012.11.001

[15] Nakamura S, Shigeyama S, Minami S, et al. LC3 lipidation is essential for TFEB activation during the lysosomal damage response to kidney injury. Nat Cell Biol. 2020;22:1252–1263. 32989250 10.1038/s41556-020-00583-9

[16] Cross J, Durgan J, McEwan DG, et al. Lysosome damage triggers direct ATG8 conjugation and ATG2 engagement via non-canonical autophagy. J Cell Biol. 2023;222(12):e202303078. 37796195 10.1083/jcb.202303078 PMC10561555

[17] Deretic V, Lazarou M. A guide to membrane atg8ylation and autophagy with reflections on immunity. J Cell Biol. 2022;221(7):e202203083. 35699692 10.1083/jcb.202203083 PMC9202678

[18] Tanida I, Sou YS, Ezaki J, et al. HsAtg4B/HsApg4B/autophagin-1 cleaves the carboxyl termini of three human Atg8 homologues and delipidates microtubule-associated protein light chain 3- and GABAA receptor-associated protein-phospholipid conjugates. J Biol Chem. 2004;279(35):36268–36276. 15187094 10.1074/jbc.M401461200

[19] Mizushima N, Noda T, Yoshimori T, et al. A protein conjugation system essential for autophagy. Nature. 1998;395(6700):395–398. 9759731 10.1038/26506

[20] Tanida I, Tanida-Miyake E, Ueno T, et al. The human homolog of *Saccharomyces cerevisiae* Apg7p is a Protein-activating enzyme for multiple substrates including human Apg12p, GATE-16, GABARAP, and MAP-LC3. J Biol Chem. 2001;276(3):1701–1706. 11096062 10.1074/jbc.C000752200

[21] Tanida I, Tanida-Miyake E, Komatsu M, et al. Human Apg3p/Aut1p homologue is an authentic E2 enzyme for multiple substrates, GATE-16, GABARAP, and MAP-LC3, and facilitates the conjugation of hApg12p to hApg5p. J Biol Chem. 2002;277(16):13739–13744. 11825910 10.1074/jbc.M200385200

[22] Tanida I, Ueno T, Kominami E. LC3 conjugation system in mammalian autophagy. Int J Biochem Cell Biol. 2004;36(12):2503–2518. 15325588 10.1016/j.biocel.2004.05.009 PMC7129593

[23] Heckmann BL, Teubner BJ, Tummers B, et al. LC3-associated endocytosis facilitates β-amyloid clearance and mitigates neurodegeneration in murine Alzheimer’s disease. Cell. 2019;178(3):536–551.e14. 31257024 10.1016/j.cell.2019.05.056 PMC6689199

[24] Fletcher K, Ulferts R, Jacquin E, et al. The WD 40 domain of ATG16L1 is required for its non‐canonical role in lipidation of LC 3 at single membranes. EMBO J. 2018;37(4):e97840. 29317426 10.15252/embj.201797840 PMC5813257

[25] Durgan J, Lystad AH, Sloan K, et al. Non-canonical autophagy drives alternative ATG8 conjugation to phosphatidylserine. Mol Cell. 2021;81(9):2031–2040.e8. 33909989 10.1016/j.molcel.2021.03.020 PMC8122138

[26] Xu Y, Zhou P, Cheng S, et al. A bacterial effector reveals the V-ATPase-ATG16L1 axis that initiates xenophagy. Cell. 2019;178(3):552–566.e20. 31327526 10.1016/j.cell.2019.06.007

[27] Hooper KM, Jacquin E, Li T, et al. V-ATPase is a universal regulator of LC3-associated phagocytosis and non-canonical autophagy. J Cell Biol. 2022;221(6):e202105112. 35511089 10.1083/jcb.202105112 PMC9082624

[28] Behrends C, Sowa ME, Gygi SP, et al. Network organization of the human autophagy system. Nature. 2010;466(7302):68–76. 20562859 10.1038/nature09204 PMC2901998

[29] Ogawa M, Yoshikawa Y, Kobayashi T, et al. A Tecpr1-dependent selective autophagy pathway targets bacterial pathogens. Cell Host Microbe. 2011;9(5):376–389. 21575909 10.1016/j.chom.2011.04.010

[30] Chen D, Fan W, Lu Y, et al. A mammalian autophagosome maturation mechanism mediated by TECPR1 and the Atg12-Atg5 conjugate. Mol Cell. 2012;45(5):629–641. 22342342 10.1016/j.molcel.2011.12.036 PMC3299828

[31] Wetzel L, Blanchard S, Rama S, et al. TECPR1 promotes aggrephagy by direct recruitment of LC3C autophagosomes to lysosomes. Nat Commun. 2020;11(1):2993. 32532970 10.1038/s41467-020-16689-5 PMC7293217

[32] Kim JH, Hong SB, Lee JK, et al. Insights into autophagosome maturation revealed by the structures of ATG5 with its interacting partners. Autophagy. 2015;11(1):75–87. 25484072 10.4161/15548627.2014.984276 PMC4502675

[33] Boyle KB, Ellison CJ, Elliott PR, et al. TECPR1 conjugates LC3 to damaged endomembranes upon detection of sphingomyelin exposure. EMBO J. 2023;42:e113012. 37409490 10.15252/embj.2022113012 PMC10476172

[34] Corkery DP, Castro-Gonzalez S, Knyazeva A, et al. An ATG12-ATG5-TECPR1 E3-like complex regulates unconventional LC3 lipidation at damaged lysosomes. EMBO Rep. 2023;24:e56841. 37381828 10.15252/embr.202356841 PMC10481663

[35] Kaur N, de la Ballina LR, Haukaas HS, et al. TECPR1 is activated by damage-induced sphingomyelin exposure to mediate noncanonical autophagy. EMBO J. 2023;42:e113105. 37409525 10.15252/embj.2022113105 PMC10476171

[36] Ellison CJ, Kukulski W, Boyle KB, et al. Transbilayer movement of sphingomyelin precedes catastrophic breakage of enterobacteria-containing vacuoles. Curr Biol. 2020;15(15):2974–2983.e6. 10.1016/j.cub.2020.05.083 PMC7416114 32649908

[37] Gao Z, Gammoh N, Wong P-M, et al. Processing of autophagic protein LC3 by the 20S proteasome. Autophagy. 2010;6(1):126–137. 20061800 10.4161/auto.6.1.10928

[38] Florey O, Gammoh N, Kim SE, et al. V-ATPase and osmotic imbalance activate endolysosomal LC3 lipidation. Autophagy. 2015;11(1):88–99. 25484071 10.4161/15548627.2014.984277 PMC4502810

[39] Thiele DL, Lipsky PE. Mechanism of L-leucyl-L-leucine methyl ester-mediated killing of cytotoxic lymphocytes: dependence on a lysosomal thiol protease, dipeptidyl peptidase I, that is enriched in these cells. Proc Natl Acad Sci USA. 1990;87(1):83–87. 2296607 10.1073/pnas.87.1.83 PMC53204

[40] Wileman T, Boshans, Schlesinger P, et al. Monensin inhibits recycling of mannose pinocytosis receptors in macrophages. Biochem J. 1984;220(3):665–675. 6087792 10.1042/bj2200665 PMC1153682

[41] Jia J, Wang F, Bhujabal Z, et al. Stress granules and mTOR are regulated by membrane atg8ylation during lysosomal damage. J Cell Biol. 2022 Nov 7;221(11). doi:10.1083/jcb.202207091. PMC9533235 36179369

[42] Yim WW-Y, Mizushima N. Lysosome biology in autophagy. Cell Discov. 2020;6(1). doi:10.1038/s41421-020-0141-7 PMC7010707 32047650

[43] Shewring JR, Hodgson L, Bryant HL, et al. Chapter 4 - Refining a correlative light electron microscopy workflow using luminescent metal complexes. In: Müller-Reichert T, Verkade P, editors Methods in Cell Biology. Vol. 162, Cambridge, Massachusetts: Academic Press; 2021. p. 69–87. 10.1016/bs.mcb.2020.12.008 33707023

