# Supplementary material for: The TECPR1:ATG5-ATG12 complex conjugates LC3/ATG8 to damaged lysosomes that expose luminal glycans in response to osmotic imbalance
Source: Autophagy Rep. 2025 May 30;4(1):2476218. doi: 10.1080/27694127.2025.2476218 (PMC12128658; doi:10.1080/27694127.2025.2476218)
Supplement: Supplemental Material [file KAUO_A_2476218_SM7768.pptx]

## Slide 1
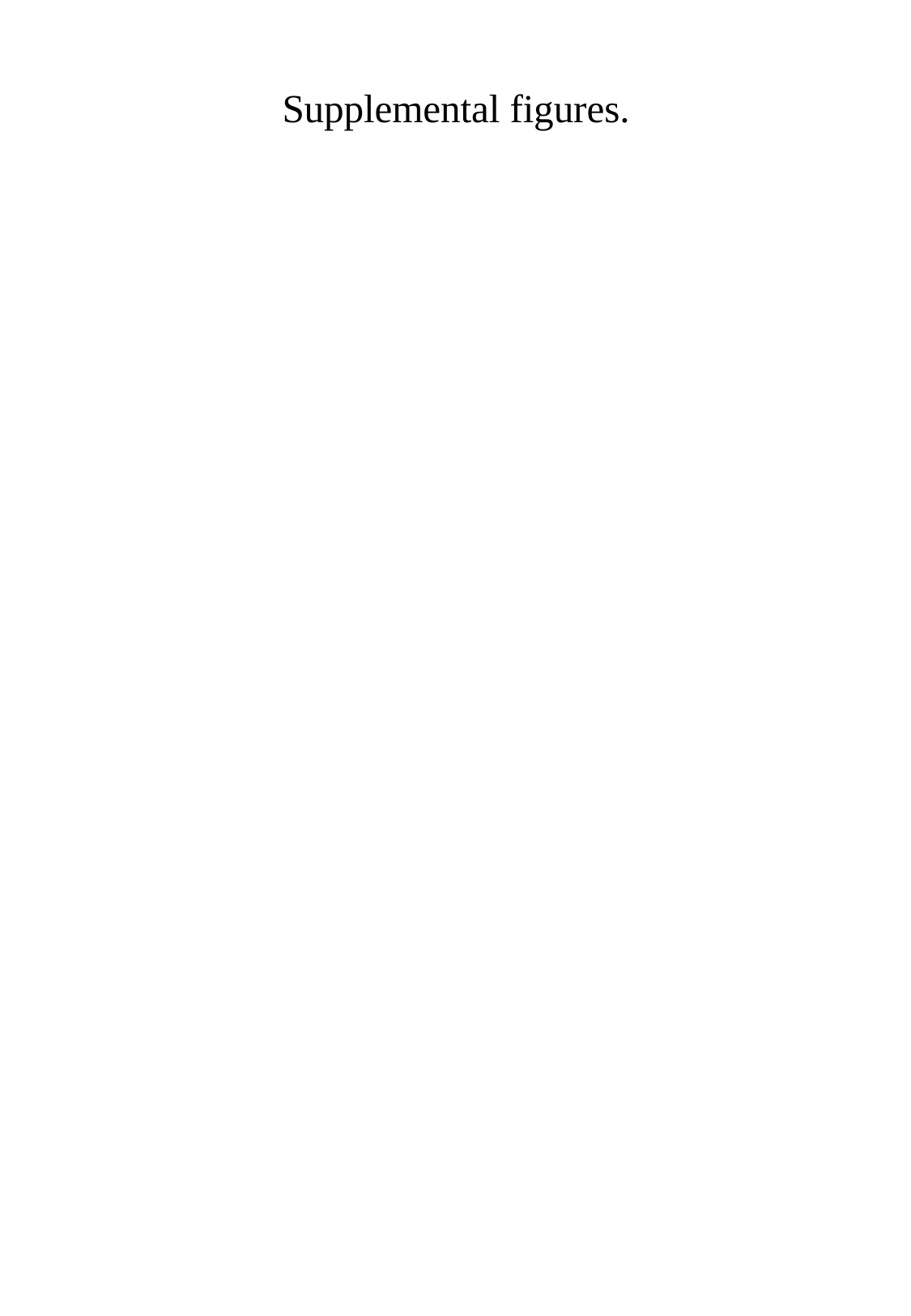

Supplemental figures.

## Slide 2
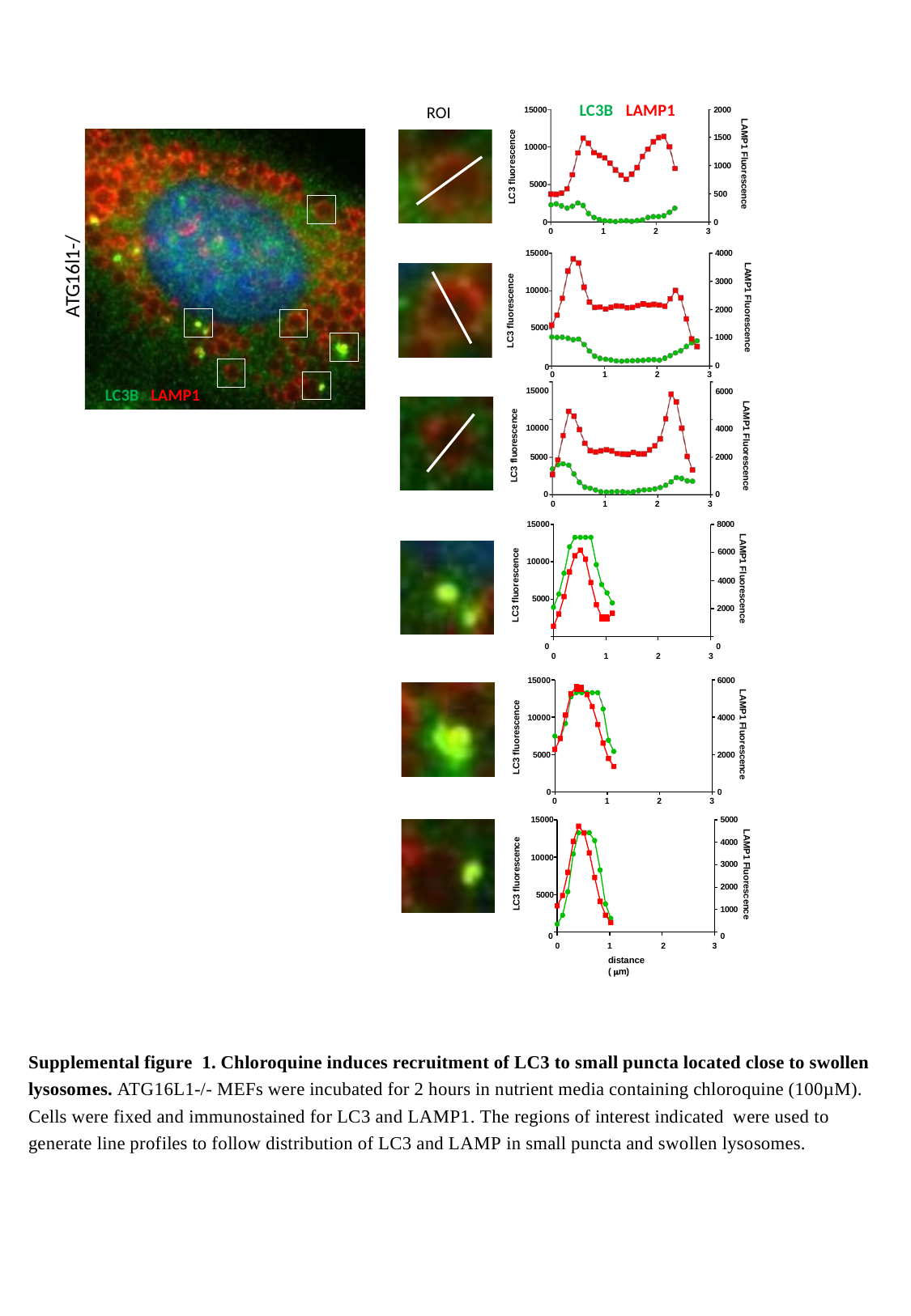

LC3B
LAMP1
ROI
15000
2000
LAMP1 Fluorescence
LC3 fluorescence
1500
10000
1000
5000
500
0
0
0
1
2
3
ATG16l1-/-
4000
15000
LAMP1 Fluorescence
LC3 fluorescence
3000
10000
2000
5000
1000
0
0
0
1
2
3
LC3B
LAMP1
15000
6000
LAMP1 Fluorescence
LC3 fluorescence
10000
4000
5000
2000
0
0
0
1
2
3
8000
15000
LAMP1 Fluorescence
LC3 fluorescence
6000
10000
4000
5000
2000
0
0
0
1
2
3
6000
15000
LAMP1 Fluorescence
LC3 fluorescence
10000
4000
2000
5000
0
0
0
1
2
3
5000
15000
LAMP1 Fluorescence
LC3 fluorescence
4000
10000
3000
2000
5000
1000
0
0
0
3
1	2
distance ( μm)
Supplemental figure 1. Chloroquine induces recruitment of LC3 to small puncta located close to swollen lysosomes. ATG16L1-/- MEFs were incubated for 2 hours in nutrient media containing chloroquine (100µM). Cells were fixed and immunostained for LC3 and LAMP1. The regions of interest indicated were used to generate line profiles to follow distribution of LC3 and LAMP in small puncta and swollen lysosomes.

## Slide 3
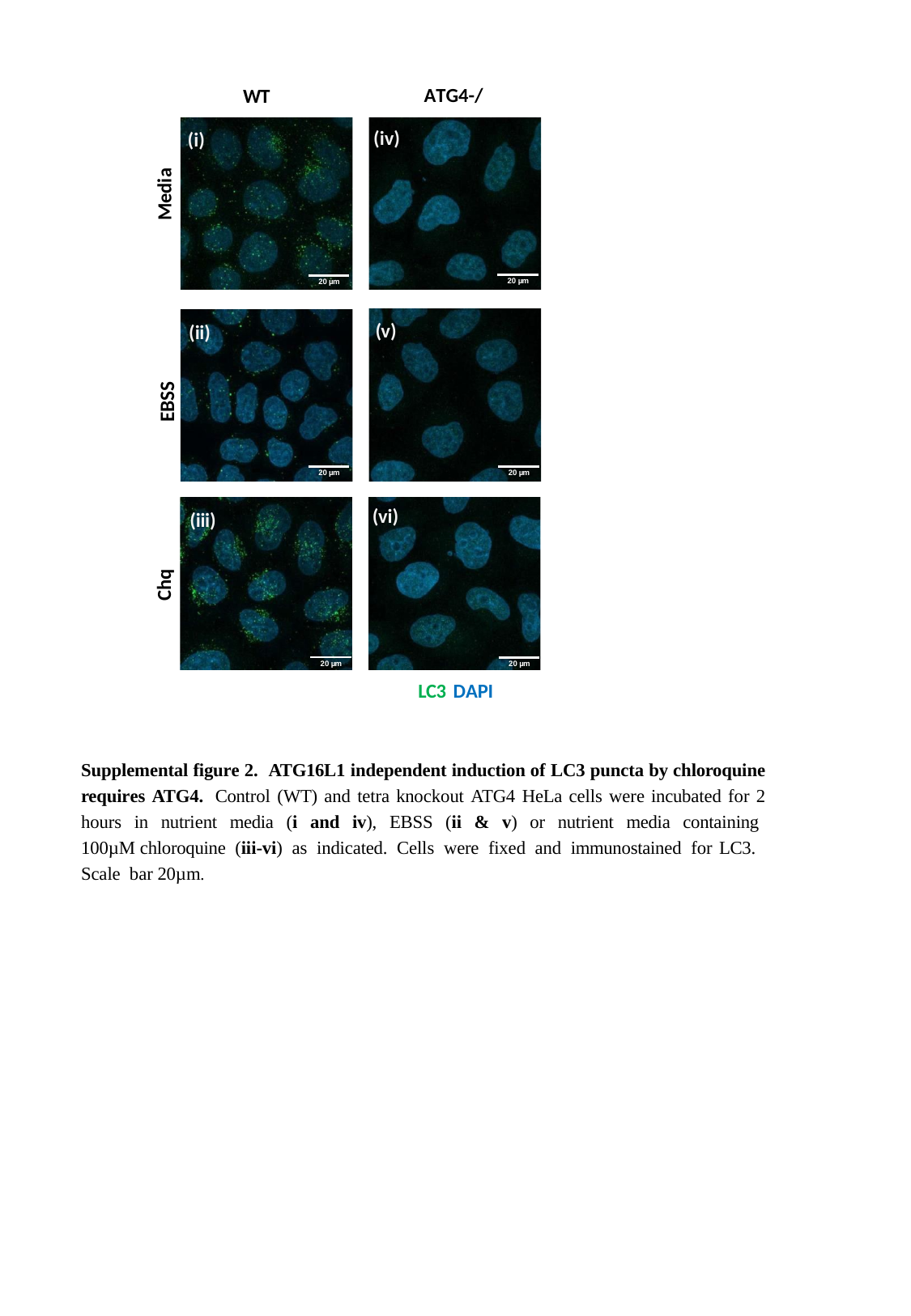

ATG4-/-
WT
(iv)
(i)
Media
20 µm
20 µm
(v)
(ii)
EBSS
20 µm
20 µm
(vi)
(iii)
Chq
20 µm
20 µm
LC3 DAPI
Supplemental figure 2. ATG16L1 independent induction of LC3 puncta by chloroquine requires ATG4. Control (WT) and tetra knockout ATG4 HeLa cells were incubated for 2 hours in nutrient media (i and iv), EBSS (ii & v) or nutrient media containing 100µM chloroquine (iii-vi) as indicated. Cells were fixed and immunostained for LC3. Scale bar 20µm.

## Slide 4
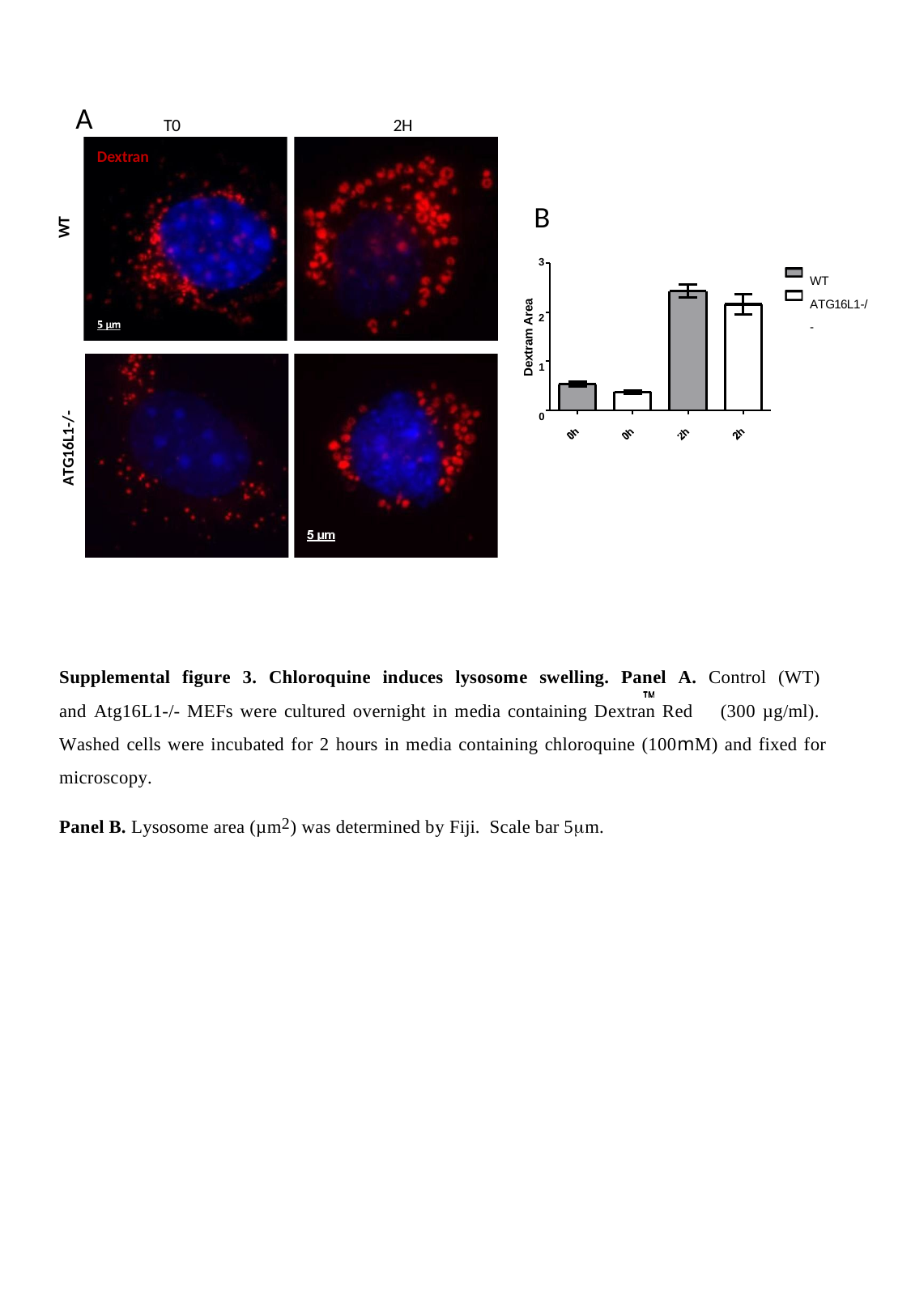

A
T0
2H
Dextran
B
WT
3
WT ATG16L1-/-
Dextram Area
2
1
0
ATG16L1-/-
Supplemental figure 3. Chloroquine induces lysosome swelling. Panel A. Control (WT) and Atg16L1-/- MEFs were cultured overnight in media containing Dextran Red (300 µg/ml). Washed cells were incubated for 2 hours in media containing chloroquine (100mM) and fixed for microscopy.
Panel B. Lysosome area (µm2) was determined by Fiji. Scale bar 5μm.

## Slide 5
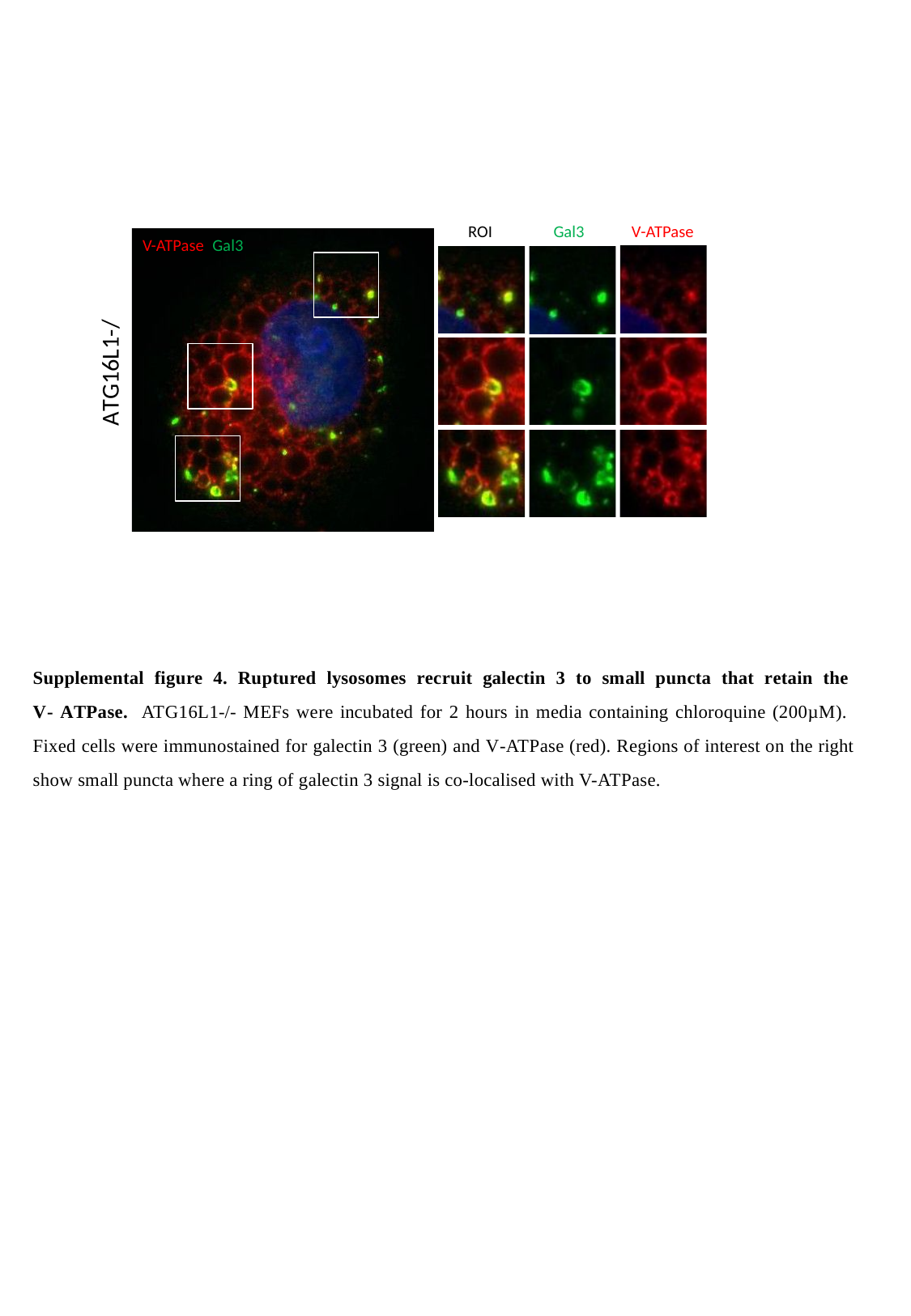

ROI
Gal3
V-ATPase
V-ATPase Gal3
ATG16L1-/-
Supplemental figure 4. Ruptured lysosomes recruit galectin 3 to small puncta that retain the V- ATPase. ATG16L1-/- MEFs were incubated for 2 hours in media containing chloroquine (200µM). Fixed cells were immunostained for galectin 3 (green) and V-ATPase (red). Regions of interest on the right show small puncta where a ring of galectin 3 signal is co-localised with V-ATPase.

## Slide 6
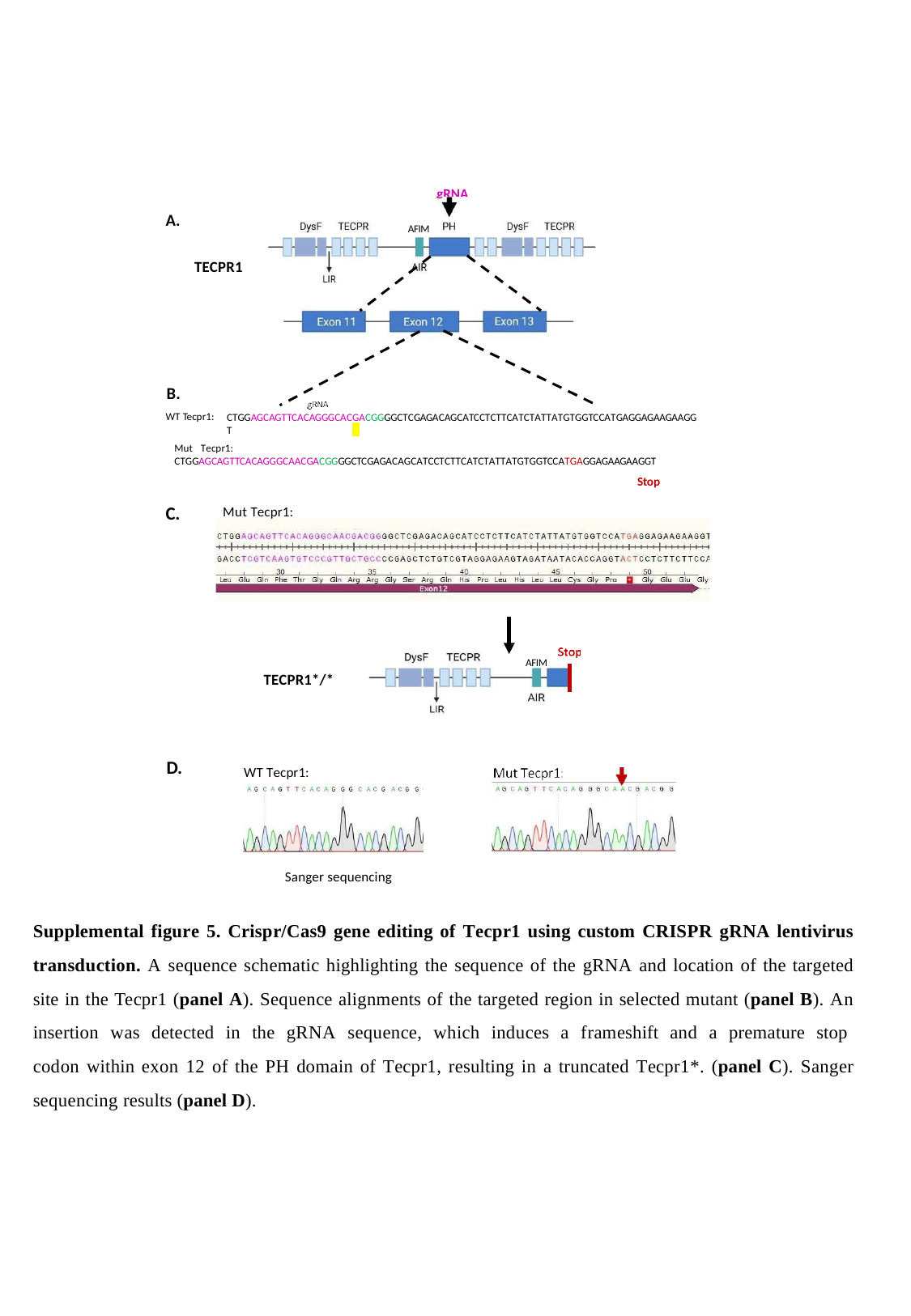

gRNA
A.
AFIM
TECPR1
B.
WT Tecpr1:
CTGGAGCAGTTCACAGGGCACGACGGGGCTCGAGACAGCATCCTCTTCATCTATTATGTGGTCCATGAGGAGAAGAAGGT
Mut Tecpr1:	CTGGAGCAGTTCACAGGGCAACGACGGGGCTCGAGACAGCATCCTCTTCATCTATTATGTGGTCCATGAGGAGAAGAAGGT
Stop
C.
Mut Tecpr1:
AFIM
TECPR1*/*
D.
WT Tecpr1:
Sanger sequencing
Supplemental figure 5. Crispr/Cas9 gene editing of Tecpr1 using custom CRISPR gRNA lentivirus transduction. A sequence schematic highlighting the sequence of the gRNA and location of the targeted site in the Tecpr1 (panel A). Sequence alignments of the targeted region in selected mutant (panel B). An insertion was detected in the gRNA sequence, which induces a frameshift and a premature stop codon within exon 12 of the PH domain of Tecpr1, resulting in a truncated Tecpr1*. (panel C). Sanger sequencing results (panel D).

## Slide 7
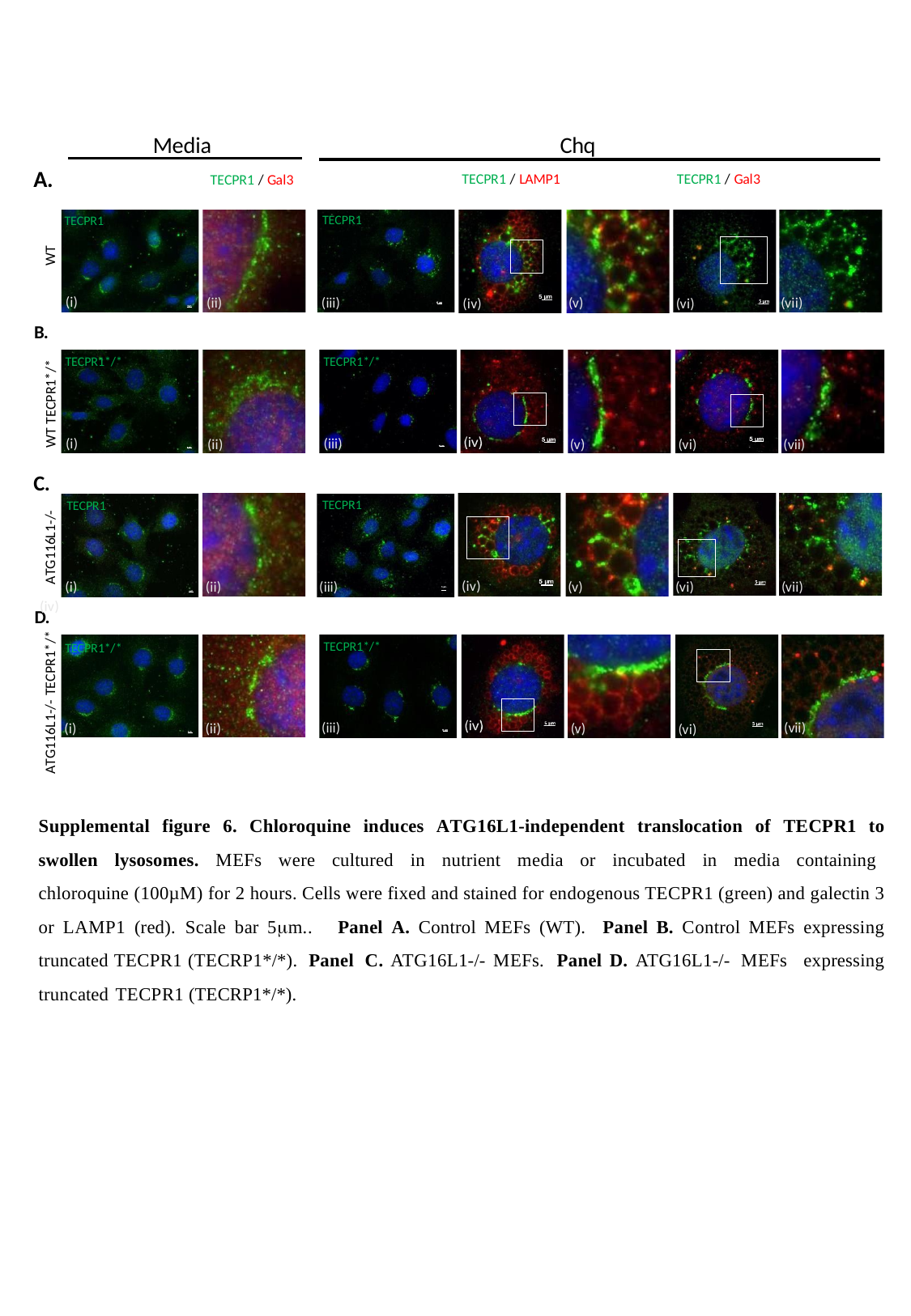

Media
Chq
A.
TECPR1 / LAMP1
TECPR1 / Gal3
TECPR1 / Gal3
TECPR1
TECPR1
WT
(i)
5 µm
(ii)
(iii)
(v)
(vii)
(vi)
(iv)
B.
TECPR1*/*
TECPR1*/*
WT TECPR1*/*
(i)
(iii)
(ii)
(v)
(vi)
(vii)
C.
TECPR1
TECPR1
ATG116L1-/-
(iv)
(iii)
(vi)
(i)
(ii)
(v)
(vii)
5 µm.
(iv)
D.
ATG116L1-/- TECPR1*/*
TECPR1*/*
TECPR1*/*
(iii)
(vii)
(i)
(ii)
(v)
(vi)
Supplemental figure 6. Chloroquine induces ATG16L1-independent translocation of TECPR1 to swollen lysosomes. MEFs were cultured in nutrient media or incubated in media containing chloroquine (100µM) for 2 hours. Cells were fixed and stained for endogenous TECPR1 (green) and galectin 3 or LAMP1 (red). Scale bar 5μm.. Panel A. Control MEFs (WT). Panel B. Control MEFs expressing truncated TECPR1 (TECRP1*/*). Panel C. ATG16L1-/- MEFs. Panel D. ATG16L1-/- MEFs expressing truncated TECPR1 (TECRP1*/*).

## Slide 8
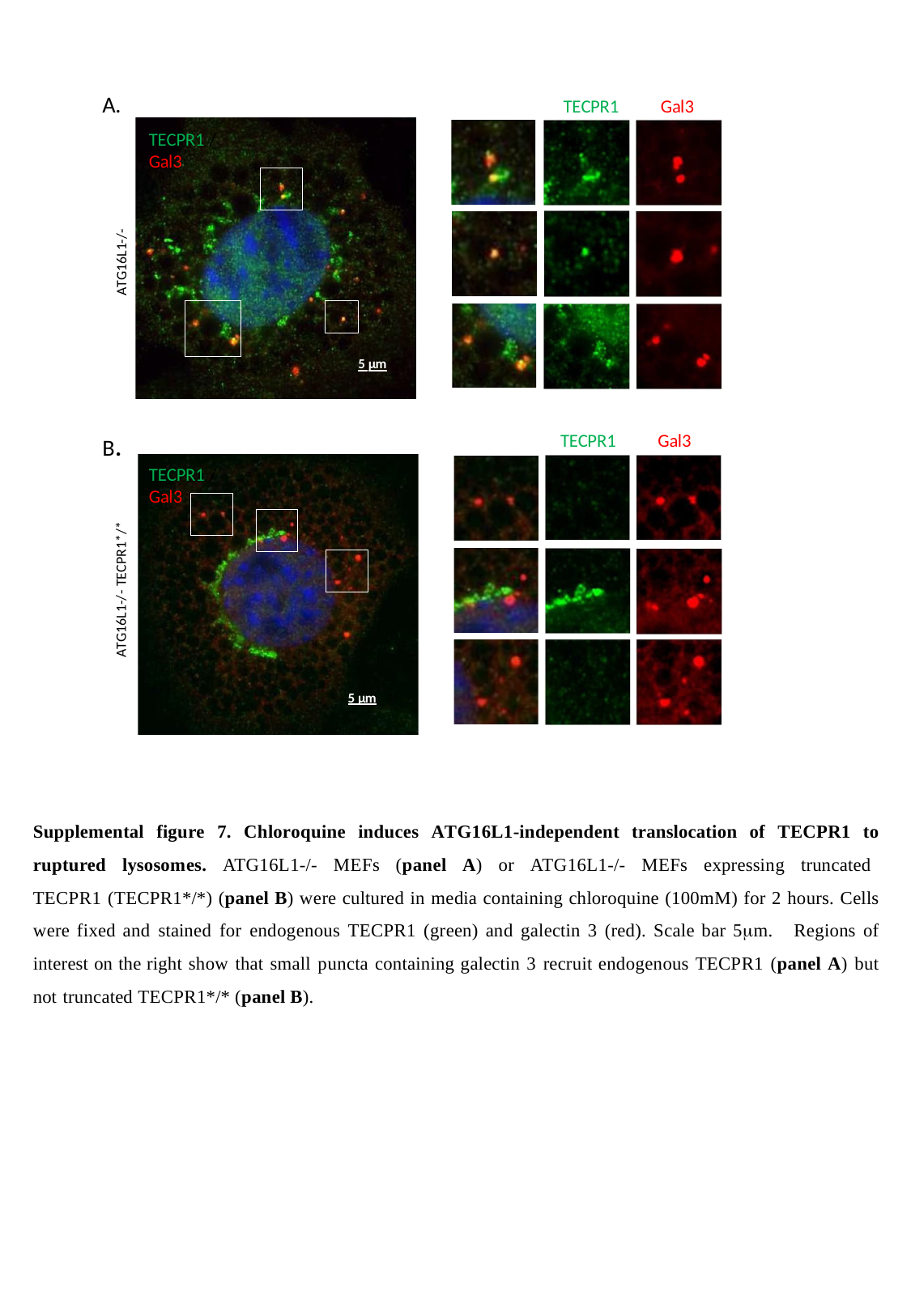

A.
TECPR1
Gal3
TECPR1 / Gal3
ATG16L1-/-
5 µm
B.
TECPR1
Gal3
TECPR1 / Gal3
ATG16L1-/- TECPR1*/*
5 µm
Supplemental figure 7. Chloroquine induces ATG16L1-independent translocation of TECPR1 to ruptured lysosomes. ATG16L1-/- MEFs (panel A) or ATG16L1-/- MEFs expressing truncated TECPR1 (TECPR1*/*) (panel B) were cultured in media containing chloroquine (100mM) for 2 hours. Cells were fixed and stained for endogenous TECPR1 (green) and galectin 3 (red). Scale bar 5μm. Regions of interest on the right show that small puncta containing galectin 3 recruit endogenous TECPR1 (panel A) but not truncated TECPR1*/* (panel B).

## Slide 9
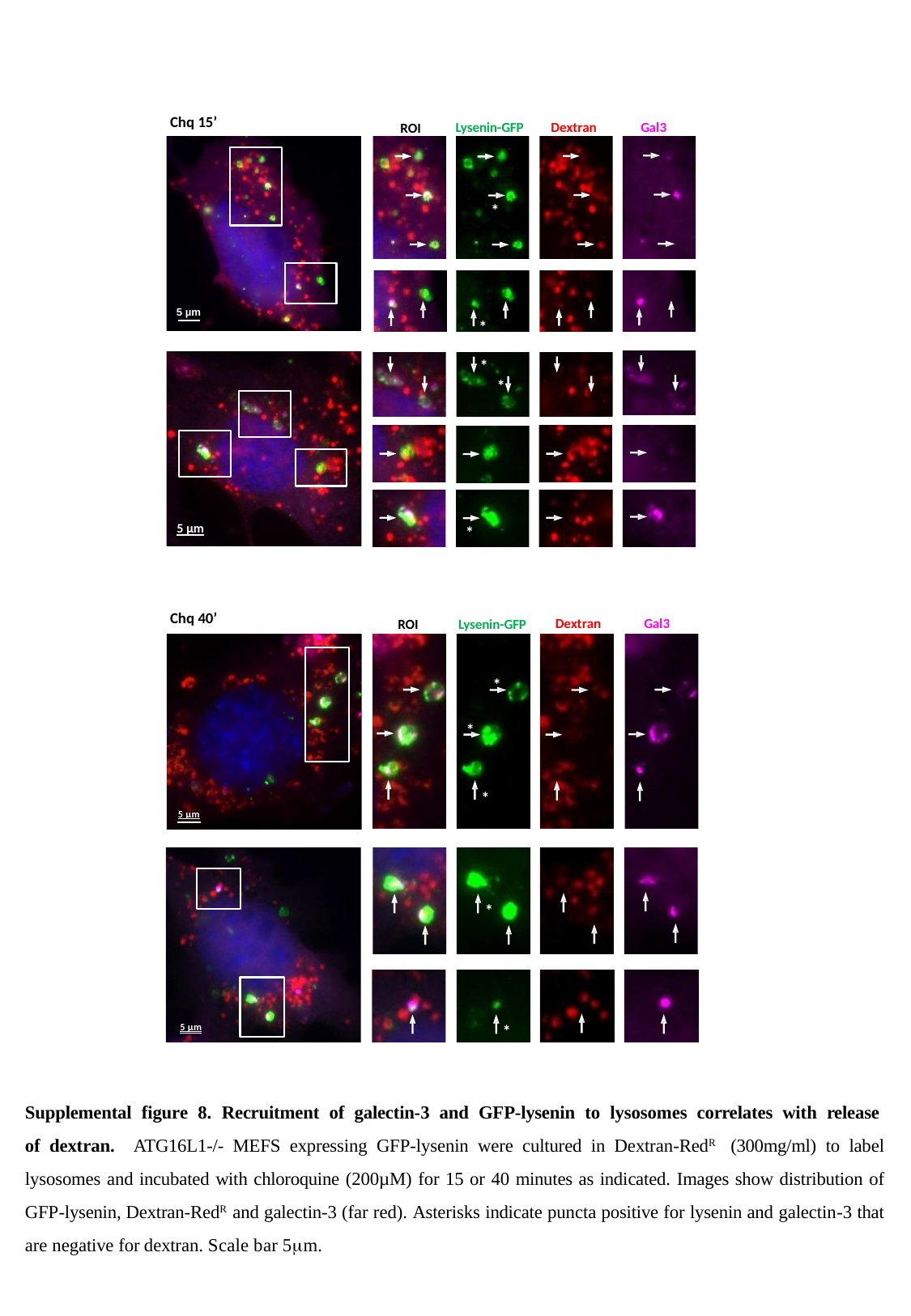

Chq 15’
Dextran
Gal3
Lysenin-GFP
ROI
*
5 µm
*
*
*
5 µm
*
Chq 40’
Dextran
Gal3
Lysenin-GFP
ROI
*
*
*
5 µm
*
*
5 µm
Supplemental figure 8. Recruitment of galectin-3 and GFP-lysenin to lysosomes correlates with release of dextran. ATG16L1-/- MEFS expressing GFP-lysenin were cultured in Dextran-RedR (300mg/ml) to label lysosomes and incubated with chloroquine (200µM) for 15 or 40 minutes as indicated. Images show distribution of GFP-lysenin, Dextran-RedR and galectin-3 (far red). Asterisks indicate puncta positive for lysenin and galectin-3 that are negative for dextran. Scale bar 5μm.

## Slide 10
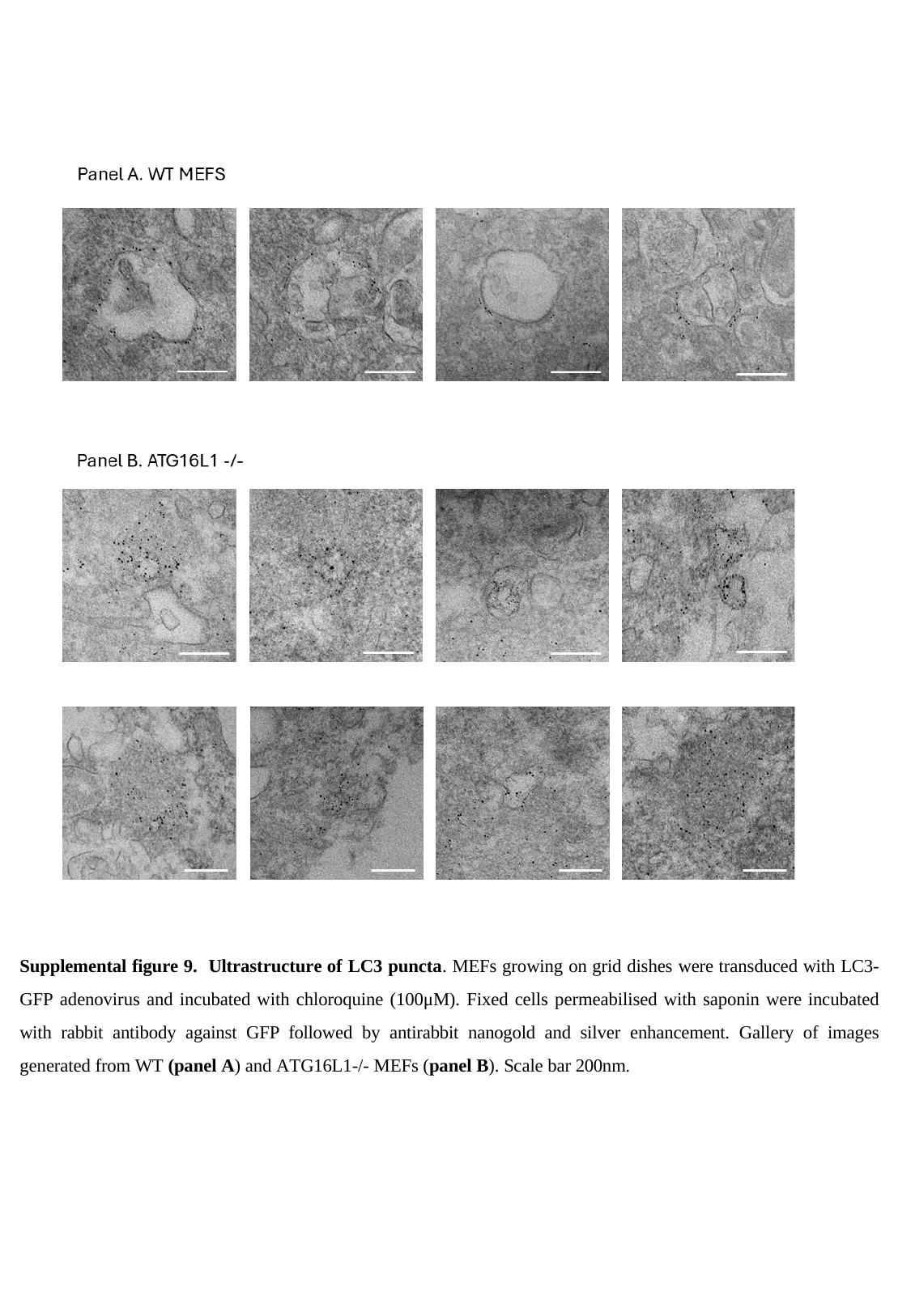

Supplemental figure 9. Ultrastructure of LC3 puncta. MEFs growing on grid dishes were transduced with LC3- GFP adenovirus and incubated with chloroquine (100μM). Fixed cells permeabilised with saponin were incubated with rabbit antibody against GFP followed by antirabbit nanogold and silver enhancement. Gallery of images generated from WT (panel A) and ATG16L1-/- MEFs (panel B). Scale bar 200nm.
